# Ga_2_O_3_ and Related Ultra-Wide Bandgap Power Semiconductor Oxides: New Energy Electronics Solutions for CO_2_ Emission Mitigation

**DOI:** 10.3390/ma15031164

**Published:** 2022-02-02

**Authors:** Zeyu Chi, Jacob J. Asher, Michael R. Jennings, Ekaterine Chikoidze, Amador Pérez-Tomás

**Affiliations:** 1 Groupe d’Etude de la Matière Condensée (GEMaC), UVSQ-CNRS, Université Paris-Saclay, 45 Av. des Etats-Unis, CEDEX, 78035 Versailles, France; zeyu.chi@uvsq.fr (Z.C.); ekaterine.chikoidze@uvsq.fr (E.C.); 2 Bay Campus, College of Engineering, Swansea University, Fabian Way, Crymlyn Burrows, Swansea SA1 8EN, UK; j.j.asher.796600@swansea.ac.uk (J.J.A.); m.r.jennings@swansea.ac.uk (M.R.J.); 3 Catalan Institute of Nanoscience and Nanotechnology (ICN2), CSIC and BIST, Campus UAB, Bellaterra, 08193 Barcelona, Spain

**Keywords:** energy electronics, ultra-wide bandgap, power electronics, diodes, transistors, gallium oxide, Ga_2_O_3_, spinel, ZnGa_2_O_4_

## Abstract

Currently, a significant portion (~50%) of global warming emissions, such as CO_2_, are related to energy production and transportation. As most energy usage will be electrical (as well as transportation), the efficient management of electrical power is thus central to achieve the XXI century climatic goals. Ultra-wide bandgap (UWBG) semiconductors are at the very frontier of electronics for energy management or energy electronics. A new generation of UWBG semiconductors will open new territories for higher power rated power electronics and solar-blind deeper ultraviolet optoelectronics. Gallium oxide—Ga_2_O_3_ (4.5–4.9 eV), has recently emerged pushing the limits set by more conventional WBG (~3 eV) materials, such as SiC and GaN, as well as for transparent conducting oxides (TCO), such asIn_2_O_3_, ZnO and SnO_2_, to name a few. Indeed, Ga_2_O_3_ as the first oxide used as a semiconductor for power electronics, has sparked an interest in oxide semiconductors to be investigated (oxides represent the largest family of UWBG). Among these new power electronic materials, Al_x_Ga_1-x_O_3_ may provide high-power heterostructure electronic and photonic devices at bandgaps far beyond all materials available today (~8 eV) or ZnGa_2_O_4_ (~5 eV), enabling spinel bipolar energy electronics for the first time ever. Here, we review the state-of-the-art and prospects of some ultra-wide bandgap oxide semiconductor arising technologies as promising innovative material solutions towards a sustainable zero emission society.

## 1. Introduction

**Figure 1:**
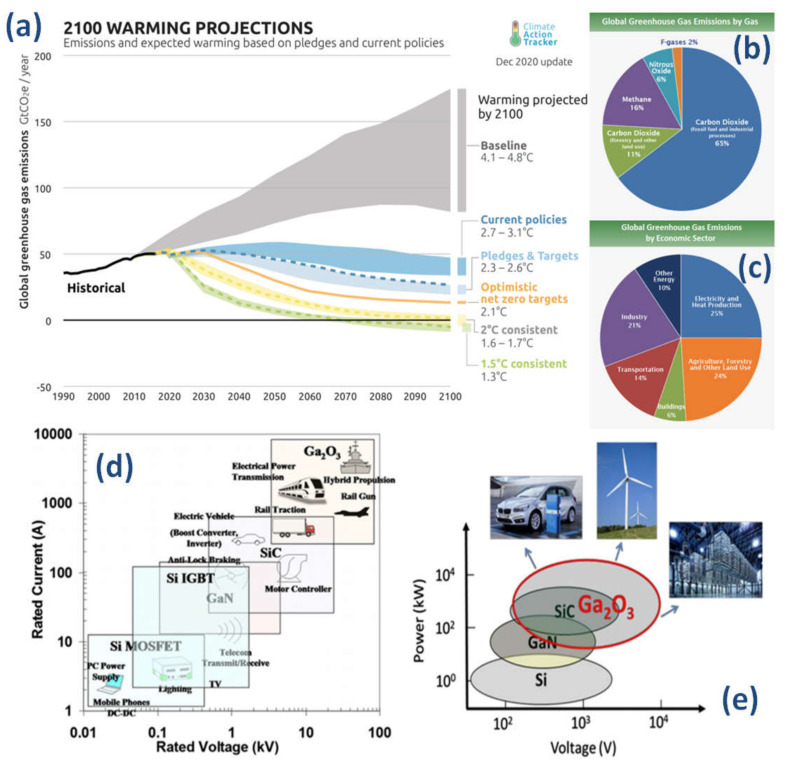
(**a**) Projected global warming figures for 2100. (**b**) Global warming emissions by gas. (**c**) Global greenhouse gas emissions by economic sector. (**d**) Selected applications for power semiconductors Si, SiC, GaN, and Ga_2_O_3_ for power electronics in terms of current and voltage requirements. (**e**) Owing to its ultra-wide bandgap, Ga_2_O_3_ can create additional possible applications for ultra-high power electronics including fast chargers for electric vehicles, high voltage direct current (HVDC) for data centers, and alternative energy sources. Figure sources: https://www.epa.gov/ghgemissions/global-greenhouse-gas-emissions-data (accessed on 16 December 2021). Source: (**a**) Source: IPCC (2014); based on global emissions from 2010. Details about the sources included in these estimates can be found in the Contribution of Working Group III to the Fifth Assessment Report of the Intergovernmental Panel on Climate Change. (**b**) IPCC (2014) based on global emissions from 2010. Details about the sources included in these estimates can be found in the Contribution of Working Group III to the Fifth Assessment Report of the Intergovernmental Panel on Climate Change. (**c**) Boden, T.A., Marland, G., and Andres, R.J. (2017). Global, Regional, and National Fossil-Fuel CO_2_ Emissions. Carbon Dioxide Information Analysis Center, Oak Ridge National Laboratory, U.S. Department of Energy, Oak Ridge, Tenn., U.S.A. doi 10.3334/CDIAC/00001_V2017. Panels (**d**) and (**e**) adapted with permission from [4](© 2018 COPYRIGHT AIP Publishing).

According to the latest Intergovernmental Panel on Climate Change (IPCC) report released in August 2021 [1], climate change is widespread, rapid, and intensifying and some trends are now regarded as irreversible. Human-induced climate change is already affecting many weather and climate extremes in every region across the globe. Scientists are also observing changes across the whole Earth’s climate system; in the atmosphere, in the oceans, ice floes, and on land. Many of these changes are unprecedented and some of the shifts are now in motion, while some—such as rising sea levels—are already irreversible for the coming centuries to millennia. Stabilizing the climate will require strong, rapid, and sustained reductions in greenhouse gas emissions, and reaching net zero CO_2_ emissions. Limiting other greenhouse gases and air pollutants, especially methane, could be beneficial for the health of the climate as well as the population [1]. The breakdown for the different greenhouse gas emissions can be seen in Figure 1 [2], where transport and electrical production account for up to 40%. Therefore, many energy-related megatrends of our modern society must focus on themes such as energy efficiency, e-mobility, smart grid and digitalization requiring green energy management electronics or power electronic solutions [3].

Around half of the power used in the world is electrical and this is expected to increase steadily in the near future [5]. The vast majority (if not all) of this electricity will flow through, at least, one power electronic device during its generation, transmission, and final use. This is a critical aspect of power management which is sometimes overlooked, as power electronics make renewable (and non-renewable) energy impactful by increasing their efficiency [6]. As Si-based devices are replaced with other materials which are more energy efficient, this will affect the overall power consumption which will have a knock-on effect on CO_2_ emissions by a significant amount [7]. Furthermore, devices made with a semiconductor having a bandgap larger than silicon can be made with less material and have lower cooling requirements, hence saving a lot of space and weight in applications such as electrical transport. This integration obviously impacts the amount of power required and, therefore, saves energy and its associated emissions. Since the 1980s, there has been a lot of work towards replacing silicon-based (E-gap of 1.12 eV) power electronics devices with wide bandgap (3–3.4 eV) semiconductor (WBG) based devices (in particular, silicon carbide (SiC) and gallium nitride (GaN)) and power devices with superior specs (higher temperature of operation, higher power handling capability, etc.) are now commercially available (typically in the range of 650 V–3.5 kV) [8,9] (Figure 1). While SiC devices and GaN transistors are already qualified in many emerging applications, silicon-based devices are still dominating in most applications. There are several reasons for this dominance, to start with, Si-based devices still have substantial potential. Their electrical and thermal performance is outstanding, their reliability is proven as can be seen from their years in application, as well as their low cost. In contrast WBG devices are starting their development, where we are still learning about materials development and device design. The benefits on the system level needs to be qualified and long-term reliability issues need to be determined; as these materials are developed, the costs for high-quality large volume production should decrease.

More recently, the frontier in the field is now given by ultra-wide bandgap semiconductors (UWBG), which have the promise of further upshifting the power rating and operation temperature. The same UWBG oxides also offer the potential for deeper ultraviolet optoelectronics [10]. Although another UWBG semiconductor, diamond, has been investigated over the last forty years, there has been limited progress and only recently have other materials, such as gallium oxide (Ga_2_O_3_) or aluminum nitride (AlN), yielded device demonstrations with appropriate performances. In particular, Ga_2_O_3_ is a newer UWBG material (4.5–5 eV) and is receiving a lot of attention as a novel semiconductor, owing to its unusual material properties. The doping (*n*-type) is very tunable with an extremely high breakdown field and unique optoelectronic properties, these alongside the possibility of growing large native substrates (over 6″) with a low cost [11]. Besides, representing the first viable oxide semiconductor for power electronics, Ga_2_O_3_ has opened the door to many more oxide compounds to be scrutinized (e.g., spinel ZnGa_2_O_4_) as they represent the largest family of ultra-wide bandgap semiconductors. UWBG oxide semiconductors are now at the very frontier of energy electronics, and much cutting-edge research, challenges, and opportunities are taking place [12]. These will be succinctly overviewed in this paper.

## 2. Oxide Semiconductors for Power Electronics

**Figure 2:**
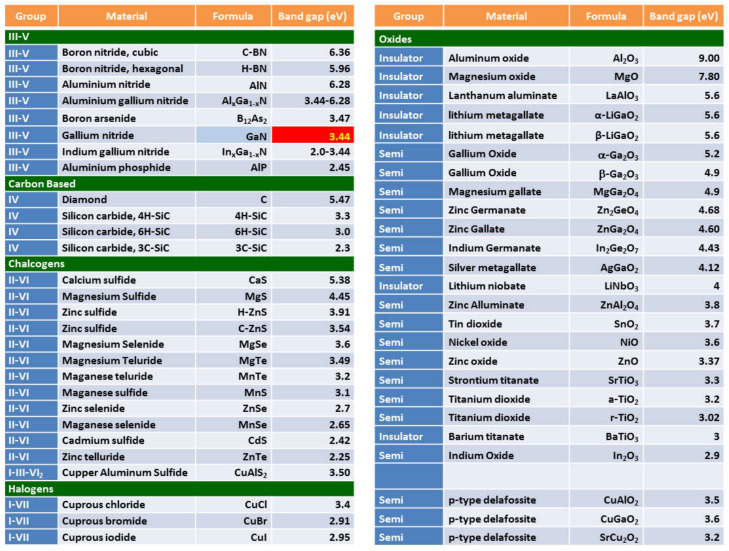
Wide bandgap semiconductors (in the context of power electronic devices) usually representmaterialswhosebandgap is larger than that of silicon. In practice, wide bandgap materials of choice have a bandgap of around ~3 eV, with silicon carbide and gallium nitride in a prominent position. Recently, a new family of semiconductor materials with even larger bandgaps (known as ultra-wide bandgap semiconductors) is being investigated for the new generation of optoelectronic and power electronic applications. As a rule of thumb, an ultra-wide bandgap semiconductor is one whose bandgap is larger than that of GaN (i.e., 3.4 eV). Perhaps the most investigated ultra-wide bandgap semiconductors are diamond, some nitrides (AlGaN, AlN, and BN), and a few oxides. Among these oxides, gallium oxide is the only oxide semiconductor with ultra-large bandgap where it is possible to modulate the conductivity (i.e., doping) to define power electronic devices.

As an alternative to silicon, there is a new generation of wide bandgap semiconductors which have the capability to operate at higher voltages, temperatures, and switching frequencies with greater efficiencies compared to existing Si devices. This characteristic results in lower losses and enables significantly reduced volume due to decreased cooling requirements and smaller passive components contributing to overall lower system cost. Wide bandgap semiconductors (in the context of power electronic devices) usually represent materials whose band gap is larger than that of silicon. A (non-exhaustive) list of different wide bandgap semiconductors is presented in Figure 2. There are several families of wide bandgap semiconductors depending on their chemical composition. The III–V wide bandgap semiconductors are primarily nitrides, phosphides, and arsenides. Chalcogen semiconductors are those containing a transition metal and a chalcogen anion (S, Se, or Te), therefore forming sulfides, selenides, and tellurides. There are few halogen wide bandgap semiconductors in the form of chloride, iodides, and bromides. Silicon carbide (which exhibits a very large number of polytypes) and diamond are both carbon-based materials. SiC is a relevant wide bandgap semiconductor since it is the only compound semiconductor that can be thermally oxidized to form SiO_2_ in the same fashion as silicon [13].

A special case of chalcogenides would be oxides; although group 16 is defined as chalcogens, the term chalcogenide is more commonly reserved for sulfides, selenides, and tellurides only. Oxides are ubiquitous in nature due to the large abundance of oxygen in the earth and the large oxygen electronegativity (i.e., the atom tendency to attract electrons and thus form bonds) that easily creates largely covalent stable chemical bonds with almost all elements to give the corresponding oxides. Indeed, almost the entire Earth’s crust parts are oxides as the individual crust elements are inclemently oxidized by the oxygen present in the atmosphere or in the water [14]. Besides, the Earth’s mantle (which represents 60–70% and ~80% of the Earth’s mass and volume, respectively) is predominantly a layer of silicate (i.e., compounds containing silicon and oxygen including silica, orthosilicates, metasilicates, pyrosilicates, etc.) and magnesium oxide (MgO)-rich rock between the crust and the outer core [14]. The upper mantle is dominantly peridotite, composed primarily of variable proportions of the minerals olivine ((Mg,Fe)_2_SiO_4_), pyroxenes (XY(Si,Al)_2_O_6_), and aluminous phases, such as feldspar (NaAlSi_3_O_8_–CaAl_2_Si_2_O_8_) and spinel (MgAl_2_O_4_). The lower mantle is composed primarily of bridgmanite ((Mg, Fe)SiO_3_) and ferropericlase ((Mg, Fe)O), with significant amounts of calcium perovskite (CaSiO_3_) and calcium-ferrite oxides [15].

Thus, in general, oxides can be regarded as naturally abundant and stable compounds. Since the early days of solid-state physics, (undoped) oxides have been considered to be insulators (or more precisely, highly resistive wide bandgap semiconductors). The bandgap of many common oxides, such as Al_2_O_3_, SnO_2_, TiO_2_, In_2_O_3_, Cu_2_O, WO_3_, ZnO, or NiO, is much wider than that of silicon (1.12 eV). Therefore, they are intrinsically poor conductors at room temperature if they are not properly doped into a degenerated state. Recently, much effort has been put into increasing the conductivity of some of these oxides (in particular those where *s* and *p* electrons propagate with a large mobility) while maintaining the optical transparency. Good examples are the doping of Al in ZnO, Sn in In_2_O_3_, and F in SnO_2_, which are known as transparent conducting oxides (TCOs).

In practice, wide bandgap materials of choice have a bandgap of around ~3 eV, with silicon carbide and gallium nitride in a prominent position. Recently, a new family of semiconductor materials with even larger bandgaps (known as ultra-wide bandgap semiconductors) is being investigated for the new generation of optoelectronic and power electronic applications. As a rule of thumb, an ultra-wide bandgap semiconductor is one with a band gap larger than that of GaN (i.e., 3.4 eV). Perhaps the most investigated ultra-wide bandgap semiconductors are diamond, some nitrides (AlGaN, AlN, and BN), and few oxides. Among oxides, gallium oxide (Ga_2_O_3_) is the only oxide semiconductor with ultra-large bandgap where it is possible to modulate the conductivity (i.e., doping) to define power electronic devices. SiC and GaN power devices have already attracted much attention in higher efficiency electrical power conversion [4]. The major advantage of *β*-Ga_2_O_3_ is that the single crystal structure can be synthesized via several standard melt growth methods, e.g., the Czochralski (CZ) technique. This is a huge advantage of Ga_2_O_3_ over SiC, GaN, and diamond for scaling up production, hence we would expect the cost of *β*-Ga_2_O_3_ power electronics to decrease and be more in line with silicon with respect to their SiC and GaN counterparts [16,17].

## 3. Gallium Oxide (Ga_2_O_3_)

Ga_2_O_3_ has, at least, six polymorphs of which only one is thermodynamically stable at high temperatures (*β* phase, monoclinic), while the others are metastable and tend to convert to *β* upon high-temperature treatments including the phases α, corundum, δ, cubic, and ε, hexagonal, γ, defective-spinel, and orthorhombic κ polymorph [18]. The basic principles of polymorphism in crystals are clear: the lattices adapt to the minimum energy with respect to the temperature and pressure. Nearly all Ga_2_O_3_-containing devices utilize the monoclinic *β* phase, the most stable and best-characterized polymorph. As a well-known representative of a binary metal-oxide, gallium oxide cannot therefore be regarded as a new material, but as a revisited and rejuvenated one. For example, early crystallographic studies for single crystals [19] together with diverse luminescence studies of doped *β*-Ga_2_O_3_ were reported as early as the1960s [20]. Lorenz et al. [21] already published in 1966 that *n*-type Ga_2_O_3_ exhibits mobilities in the range of 100 cm^2^V^−1^s^−1^ and an adequate device doping of 10^18^ cm^−3^ can be achieved just by controlling the native oxygen vacancies’ density. Its deep-ultraviolet intrinsic bandgap of around 4.5–4.9 eV and excellent photoconductivity are also well-known from early contemporary studies [22]. It was not until this decade that the potential of Ga_2_O_3_ for a certain class of extreme or power electronics was realized due to further availability of large-area single crystals with high quality and the control of doping. In the past, Ga_2_O_3_ was somehow ignored as an ultra-wide bandgap material, as it was eclipsed by the potential of diamond which has never been fully realized [23].

Previously, SiC and GaN were the wide bandgap materials of choice [6]. However, from an ultra-high energy electronics perspective, Ga_2_O_3_ transistors and diodes exhibit the potential of delivering outstanding performances in the form of high breakdown voltage, high power and low losses because of superior material properties, thus extending the power handling limits given by the SiC and GaN integration into the mainstream [4]. Indeed, an ultra-large breakdown electric field, (which is usually assumed to be of the order of *E_c_*~8 MVcm^−1^), is a prime material advantage of Ga_2_O_3_. However, this value may be well underestimated; it was very recently suggested that the critical electric field of Ga_2_O_3_ could be as large as 13.2 MVcm^−1^, if the residual donors are efficiently removed [24].

**Figure 3:**
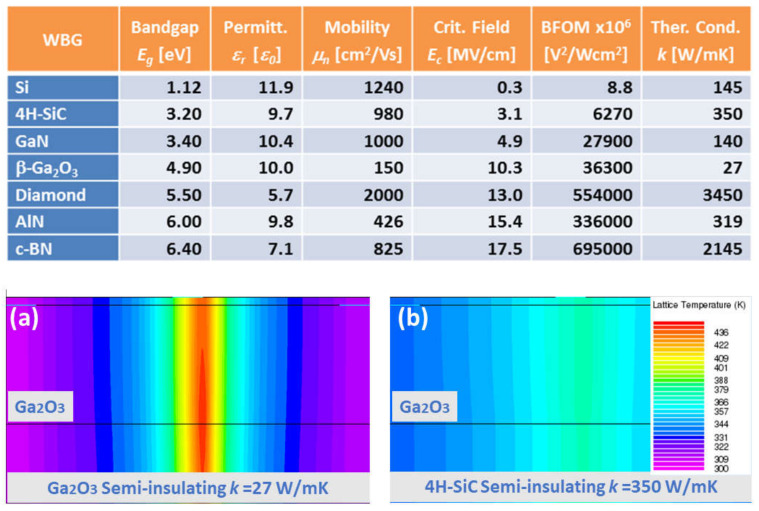
A summary of the main power device figure of merit (or Baliga’s figure of merit. BFOM) parameters of the most popular wide bandgap semiconductors. Gallium oxide has a particularly poor thermal conductivity. However, when integrated into devices, heterojunctions with other better suited heat sinks (such as silicon carbide) area way to circumvent that limitation. As shown in the bottom panels, the simulate lattice temperature is lower on SiC (**b**) when compared with Ga_2_O_3_ substrates (**a**). Furthermore, thinning the Ga_2_O_3_ active film helps thermal performances. Adapted with permission from [11] © 2018 COPYRIGHT Society of Photo-Optical Instrumentation Engineers (SPIE).

A high critical field crucially promotes the suitability of a semiconductor material for power devices that would be able to manage a large amount of electrical energy per unit area. Baliga’s figure of merit [25] for power electronics is proportional to *E_c_^3^*, whilst only being linearly proportional to the bulk electron mobility (*µ*). Although Ga_2_O_3_ presents a similar conduction band dispersion (i.e., effective mass) than GaN, a relatively small bound limit of *µ* ~300 cm^2^V^−1^s^−1^ is frequently given [26]. This is due to a massive Fröhlich interaction which is common to many conducting oxides. Balancing critical field and mobility, the on-state losses can be still an order of magnitude lower than those for SiC and GaN for a given breakdown voltage (Figure 3). Comparing these values to other power semiconductors (see Figure 3), *β*-Ga_2_O_3_ appears favorable, surpassing SiC and GaN. A major additional technological advantage of the *β*-Ga_2_O_3_ is that the single crystal structure can be synthesized via several standard melt growth methods including the Czochralski (CZ) technique [27]. This, in practice, would imply SiC performances (or better ones) at a fraction of cost.

There are certain applications, such as maritime and air transport, that are difficult to electrify as the power ratings are generally larger than, say, urban electric cars (Figure 1d,e). For electric cars, devices delivering at or below the 1.2 kV perform well as rapid chargers or drive converters. These power ratings are well covered with “conventional” WBG, such as SiC and GaN. As the critical electric field of Ga_2_O_3_ has been reported to be at least two times, (or even four times larger), than that of these WBGs, the blocking voltage range of single electronics devices may be significantly extended in the future beyond what is theoretically possible today. These promises will impact directly on the size and weight of planes and ships resulting in less energy and emissions. As energy and transportation represents a major portion of the current CO_2_ emissions contributing to global warming, it is expected that UWBG such as Ga_2_O_3_ may open new opportunities in sectors that are now difficult to decarbonize. Other prominent examples where the advantage of ultra-wide bandgap semiconductors can be exploited are as more solar-blind (UV transparent) transparent conducting electrodes [11] and electron (or hole) transport layers within solar cells or photodiodes [28].

### 3.1. Gallium Oxide Bulk Crystal Growth

Commonly used growth techniques of bulk *β*-Ga_2_O_3_ crystal are (Table 1): Verneuil method [21,29], Czochralski (CZ) method [30,31,32,33], floating-zone (FZ) method [34], edge-defined film fed (EFG) method [16,17], and Bridgman (horizontal or vertical, HB and VB) method [35,36],summarizing the basic features of melt growth methods reported so far.

The Verneuil method, being a crucible-free technique, enables both oxidizing and reducing of growth conditions [21]. The synthesis under a reducing condition benefited electron conductivity [49]. *N*-type doping was realized by Harwig et al. [37], the free carrier concentration was determined to be ~10^19^ cm^−3^ by Mg doping, and ~10^21^ cm^−3^ by Zr doping at 900 °C. The *β*-Ga_2_O_3_ bulk crystal grown by this method has poor quality, and it was used mainly last century, as other more efficient techniques were well developed. The FZ method is also a crucible-free technique, it was recently used to grow bulk *β*-Ga_2_O_3_ crystal to investigate the scintillation features [50,51] as it can be employed in an air atmosphere, which may allow for creation of fewer oxygen defect centers being the emission origin of Ga_2_O_3_ [52]. Tomioka et al. [41] analyzed the residual impurities of *β*-Ga_2_O_3_ grown by the FZ method by inductively-coupled plasma mass spectroscopy; besides Si or Sn, Al, Mg, and Fe have also been detected with a concentration of ~10^16^ cm^−3^. Al was presumed to be a neutral impurity, while Mg and Fe were considered as deep ionized acceptors and could compensate Si donors. To our knowledge, the lowest FWHM reported is ~22 arcsec for the peak *β*-Ga_2_O_3_ (400) by Hossain et al. [39], in this work, the Laue diffraction patterns also confirmed that the grown *β*-Ga_2_O_3_ crystal has a good crystallinity. However, FWHM of *β*-Ga_2_O_3_ rocking curves larger than 100 arcsec has also been measured [38,53]. However, both these techniques mentioned above suffer from small crystal size (wafer is no more than 1 inch so far, as summarized in Table 1.

Using an Ircrucible, the CZ method has been predicted to be a potential candidate for large boule, but thermal instability is an issue at high temperature that leads to decomposition of Ga_2_O_3_. Thus, this technique requires atmosphere control. Being a crack-free technique, the *β*-Ga_2_O_3_ crystal grown by the CZ method has small or even no boundaries. Several works reported by Galazka et al. [32,43,44] evidenced that the FWHM of the X-ray rocking curve could be as low as 22–50 arcsec on average, and the dislocation density was ~103 cm^−2^. Moreover, Galazka et al. [31] recently reported that bulk Ga_2_O_3_ grown by the CZ method has an electron mobility of 80–152 cm^2^V^−1^s^−1^ with a low residual Si impurity concentration of ~10^16^ cm^−3^. Similar to the CZ method, the EFG method has the same technique issue. However, this technique is available for a 4-inch wafer and recently became commercially available. Commonly observed twin-boundaries in the EFG grown *β*-Ga_2_O_3_ were efficiently avoided by optimizing the growth process (the so-called shouldering process). Different from the traditional growth direction (010), Oshima et al. [54] demonstrated that the (001) oriented *β*-Ga_2_O_3_ grown by the EFG is more suitable than (010) for a Schottky barrier diode (SBD). A weak correlation between pits and electrical properties has been revealed [27,54]. The use of the VB method allows withstanding of high oxygen concentrations as a Pt-Rh (70–30%) alloy crucible. Additionally, this crucible also facilitates the pulling-up process as the grown crystal does not adhere to the wall. The major residual impurities are generally Rh (~several tens wt.ppm) from the crucible, Sn and Si (~several wt.ppm) from raw materials, and Fe and Zr (~several wt.ppm) from the furnace [36,48].This technique recently became *n*-type doping available by using a resistance heating VB furnace, and electron concentration and electron mobility were determined to be 3.6 × 10^18^ cm^−3^ and 60 cm^2^V^−1^s^−1^, respectively, by 0.1 mol% Sn-doped [35,48]. As the CZ, EFG, and VB method use the crucible, they all have a high level of scalability.

### 3.2. Gallium Oxide Thin-Film Growth

Bulk devices and subsequent epitaxy of *β*-Ga_2_O_3_ layers could be provided by bulk growth, while high-quality epitaxial growth technologies are still required in order to study and fabricate more complex devices. Halide vapor phase epitaxy (HVPE), metal-organic vapor phase epitaxy (MOVPE), pulsed laser deposition (PLD), atomic layer deposition (ALD), molecular beam epitaxy (MBE), mist-chemical vapor deposition (CVD), and metal-organic chemical vapor deposition (MOCVD) are all involved in thin-film growth of Ga_2_O_3_.

Vapor phase epitaxy is a commercially promising technique for mass production of *β*-Ga_2_O_3_. Based on VPE, the halide vapor phase epitaxy (HVPE) method enables a growth rate as high as 250 μm/h [55] and the wafer size from 2 to 6 inches [56], it is thus a suitable technique for thick films with high purity for high voltage vertical switching devices. Furthermore, with the presence of chlorine catalyst in the growth chamber, this technique exhibits the growth of metastable phases of Ga_2_O_3_, such as α and *ε* [57]. The HVPE method suffers from a high level of roughness on the surface even at a relatively low growth rate [56,58]; an electrical mechanical [59] or a chemical mechanical [60] polishing can be employed to remove further deep surface pits formed during the growth. Leach et al. [61] reported a vast difference in surface morphology and XRD full-width half-maximum (FWMH), between sufficiently and insufficiently CMP polished (discriminated by the polishing times of the various polishing steps) *β*-Ga_2_O_3_ wafers grown by HVPE. Despite the poor morphology, the FWHM of the films grown on on-axis substrate were as narrow as 28 arcsec. Moreover, Murakami et al. [62] revealed that effective donor concentration without intentional doping could reach as low as 10^13^ cm^−3^.

Metal-organic vapor phase epitaxy (MOVPE)can provide a highly scalable growth as its deposition areas are large. Triethylgallium (TEGa), trimethylgallium (TMGa), and O_2_are most commonly the precursors for gallium and oxygen, respectively. The homoepitaxial growth of *β*-Ga_2_O_3_ by MOVPE can be strongly affected by substrate orientation. The growth rate is approximately 1.6–2.0 nm/min on the (100) plane, 0.65–1 µm/h on the (010) plane, and 1.6–4.3 nm/min on the (00-1) plane with miscut angles [63]. Recently, the growth rate can be elevated to 3.6 nm/min on the (100) plane [64] by tuning the growth pressure. A high-quality homoepitaxial growth on *β*-Ga_2_O_3_ the (100) with an FWMH of 43 arcsec has been reported by Gogova et al. [65]. The study of residual donor source is still in progress [66] while an electron concentration of 8 × 10^19^ cm^−3^ by Si-doping was realized by Baldini et al. [67], which is the highest doping level by this technique so far.

Pulsed laser deposition (PLD) has often been used for doped layers of Ga_2_O_3_ as it can transport materials from the target to the substrate stoichiometrically, thus the thickness of layers can be incisively controlled. It also has a relatively low operating temperature compared to other techniques. However, the quality of the materials deposited and the deposition rate are relatively low compared with other CVD and MBE methods. The roughness measured on the surface of Ga_2_O_3_ films had a root mean square between 1 and 7 nm [68,69,70].

A growth rate of 10.8 nm/min could be reached without oxygen, while it decreased to 6.5 nm/min by increasing oxygen pressure to 50 mbar [71]. Indeed, oxygen partial pressure and temperature are considered as the dominant parameters for properties of materials grown by the PLD [72]. The crystallinity was enhanced by increasing oxygen pressure at either low deposition temperature (250 °C [71]) or high deposition temperature (780 °C [68]). A higher oxygen partial pressure also leads to self-trapped holes at O1*s* and between two O2*s* sites [68], which could further act on the transport properties. Unlike the influence of oxygen pressure, a higher temperature does not always lead to a better film quality [73,74]. While, as expected, a higher annealing temperature could improve the crystallinity, as it helps the re-arrangement of Ga and O atoms to their optimal sites [75,76]. The highest *n*-type doping level achieved by the PLD is 1.7 × 10^20^ cm^−3^ by Si doping [69].

Atomic layer deposition (ALD), initially called atomic layer epitaxy (ALE), is a sub-set of the chemical vapor deposition (CVD) technique based on self-saturation, sequential surface reactions. ALD is a more general deposition containing ALE and molecular layering (ML) techniques [77]. The highly controlled thickness of films and conformal coverage are the main advantages of ALD over other techniques, it also allows a relatively lower deposition temperature compared to MBE and CVD techniques and a lower growth rate (generally less than 0.1 nm/cycle). Sn-doped Ga_2_O_3_ grown by ALD was investigated by Siah et al. [78], however the concentration of Sn was estimated as 2 × 10^20^ cm^−3^, with the free electrons determined to be 4 × 10^18^ cm^−3^. This was due to the low growth temperature.

Thus, post-annealing is generally also required to improve the crystalline quality. Additionally, the temperature during growth depends mainly on the gallium precursor chosen [79,80]. Besides the conventional ALD, the plasma-enhanced atomic layer deposition (PEALD) further permits a lower deposition temperature and better Ga_2_O_3_film properties with very smooth surface roughness (<1 nm) [81,82,83].

Molecular beam epitaxy (MBE)suits research purposes better than commercial use, as it enables the growth of high structural quality *β*-Ga_2_O_3_ with a relatively low growth rate (<1 μm/h) and high production cost, while high voltage vertical devices often require thick drift regions (dozens of microns). The orientation of growth has been found to be one factor that influences the growth rate [84]. Mazzolini et al. [85] further demonstrated the growth rate of different orientations Γ(010) (2.3 nm/min) > Γ(001) > Γ(−201) > Γ(100) of In-catalyzed *β*-Ga_2_O_3_layers;this phenomenon was believed to be associated with the surface free energy related to the binding energy of the In ad-atom. Nepal et al. [86] reported a heteroepitaxial growth on SiC with (−402) having a relatively high FWMH (694 arcsec), which can be reduced to 30–60 arcsec by homoepitaxial growth [87]. The thin films grown by MBE also benefit a smooth surface with a roughness of less than 1 nm [88,89]. The densities of the threading dislocation etch pits was determined to be ~10^5^ cm^−2^ for the film grown at 850 °C [89]. An electron concentration of 10^20^ cm^−3^ has been achieved by Sn doping [90].

Techniques based on chemical vapor deposition (CVD) have also been employed for the growth of Ga_2_O_3_. Scalability and mass production are the most advantageous characteristics of the mist-CVD technique, as it is a vacuum free, low-cost, and solution-processed approach. This technique is also often used for epitaxial growth of α-Ga_2_O_3_ on sapphire [91,92,93,94]. Morimoto et al. [94] also pointed out the facilities of mist-CVD for Ga_2_O_3_by F doping. Both homoepitaxial [95,96] and heteroepitaxial [97] growth of *β*-Ga_2_O_3_have been successfully performed. It is also worth noting that the FWMH of rocking curves was 39–91 arcsec for homoepitaxial growth with growth rate of 0.5–3.2 µm/h [96,98]. An electron concentration was measured as 5 × 10^20^ cm^−3^ by Sn doping [98].

**Figure 4:**
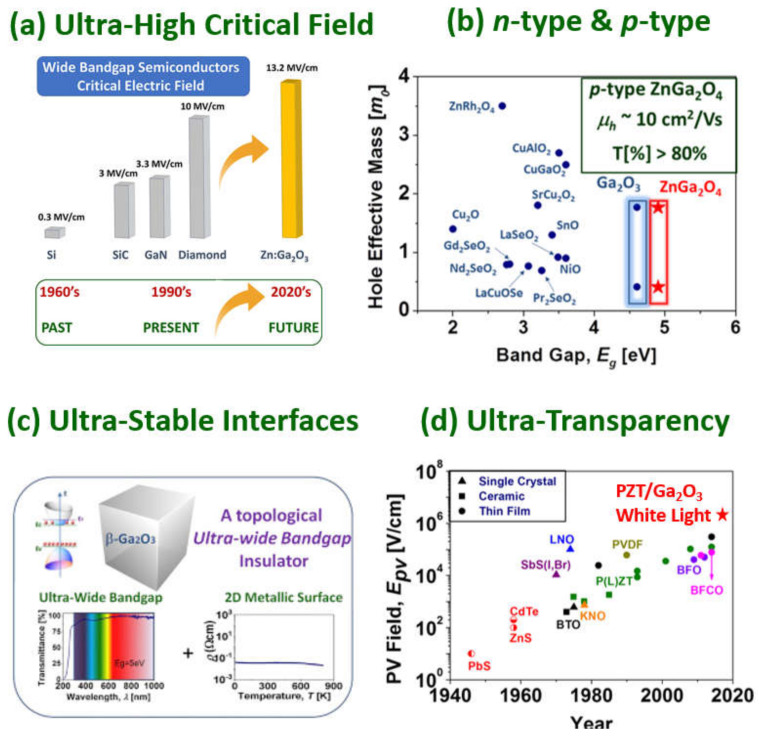
Ga_2_O_3_ and related oxides have been demonstrated to exhibit some remarkable features, such as (**a**) ultra-high critical electric field, (**b**) potential bipolar operation due to its demonstrated *n*-type and *p*-type conductivity, (**c**) ultra-stable interfaces that may host a 2D electron gas, (**d**) extended transparency into the UV-A region for transparent conducting oxide (TCO) applications (tail state density is located deeper in the ultraviolet than conventional TCOs). Panel (**a**) adapted with permission from Chikoidze et al. [24] © 2022 Elsevier Ltd. All rights reserved. Panel (**b**) adapted with permission from Chikoidze et al. [106] Copyright © 2022, American Chemical Society. Panel (**c**) adapted with permission from Chikoidze et al. [107]. © 2022 Elsevier Ltd. All rights reserved. Panel (**d**) adapted with permission from Perez-Tomas et al. [108,109] © 2022 WILEY-VCH Verlag GmbH & Co. KGaA. Adapted with permission from [12] © 2021 COPYRIGHT Society of Photo-Optical Instrumentation Engineers (SPIE).

The metal-organic chemical vapor deposition (MOCVD) technique uses Ga-based organic material as metal precursors, such as trimethylgallium (TMGa) and triethylgallium (TEGa), which usually leads to C-contamination of the as-grown film (relatively less carbon by using TEGa than TMGa). It is well-known that such contamination can be efficiently reduced by high growth temperature, and eliminated by post-annealing. Li et al. [99] reported a high-quality homoepitaxially grown film with FWMH and surface roughness of 21.6 arcsec and 0.68 nm, respectively. The growth rate is generally from several hundred nm/h [100,101] to10 µm/h [102,103,104]. This technique is also available for both *n*- and *p*-type dupability [24,105] (Figure 4).

### 3.3. Gallium Oxide Doping Issues and Recent Progress

*β*-Ga_2_O_3_ is very easily doped *n*-type to the degenerate state, *n*-type doped *β*-Ga_2_O_3_ with carrier concentration from 10^16^ to 10^20^ cm^−3^ [110,111] has been achieved by Sn and Ge doping by MBE, Si and Sn doping by MOVPE, and Sn doping by MOCVD [69]. A high mobility at room temperature of 145–184 cm^2^V^−1^s^−1^ [100,101,112] has been reached by Si doping, and even till 10^4^ cm^2^V^−1^s^−1^ at 46 K [109]. Having a high critical field (5.2 MV.cm^−1^ without intentional doping [113]), the *β*-Ga_2_O_3_devices demonstrate high performance. Nevertheless, all the Ga_2_O_3_devices demonstrated thus far have been unipolar in nature (i.e., only *n*-type). In order to realize the full potential for WBG opto-electronics *β*-Ga_2_O_3_and to sustain high breakdown voltage (>6.5 kV), we need vertical geometry bipolar-junction-based devices. Therefore, the realization of *p*-type *β*-Ga_2_O_3_ is a primary challenge today for the gallium oxide scientific community (Figure 4).

There is a tendency in oxide compounds to have *n*-type conductivity, caused by vacancies in the oxygen atoms. This, as well as the fact that it is a UWBG material, intrinsic conduction is rare and even causes *p*- and *n*-type doping tends not to be symmetrical. This asymmetry is seen in gallium oxide, the hole conductivity is poor and is likely the main limitation for development of gallium oxide technology. Fundamental restrictions such as this area recurring issue in oxides, such as: (i) acceptor point defects with high formation energy; (ii) native donor defects with low energy—resting holes; and (iii) *p*-type oxides suffer from a high effective mass of the holes (this results in a low mobility), due to the top of the VB predominantly from localized O 2-p derived orbits.

Native *p*-type conductivity: Using thermodynamical calculations for the point defects on gallium oxide it can be seen that gallium oxide is “lucky”, as when *β*-Ga_2_O_3_is at 500 °C, *P_hole_* ≈ 1.33 × 10^−2^ atm with a hole concentration around *p* ≈ 10^15^ cm^−3^ [114]. Comparing this to calculations for ZnO gives *P_hole_* ≈10^3^ atm, for the same temperature. This divergence is believed to be from higher formation energy of the donor vacancies in *β*-Ga_2_O_3_ (approximately 1 eV higher per vacancy), making compensation mechanism by point defects less favorable in gallium oxide than in ZnO. As a consequence, it can be expected that *p*-type samples of *β*-Ga_2_O_3_ with higher carrier concentrations (then intrinsic) can be obtained when doping with shallow acceptor impurities.

The native hole concentration was investigated by Nanovation (SME, France) [114] where undoped *β*-Ga_2_O_3_thin film grown on c-sapphire substrates by pulsed laser deposition (PLD) showing resistivity of *ρ* = 1.8 × 10^2^ Ω.cm, hole concentration of *p* = 2 ×10^13^ cm^−3^ and a hole mobility of 4.2 cm^2^V^−1^s^−1^ [114]. The determination of conductivity mechanism showed that Ga vacancies act as deep level acceptors with the activation energy of 0.56 eV in the low compensated sample, having *Ea* = 1.2 eV ionization energy. Later, the improvement was shown that native *p*-type conductivity by post-annealing in an oxygen atmosphere for *β*-Ga_2_O_3_ thin film was grown on c-sapphire substrates by MOCVD [115]. After oxygen annealing, the hole concentration was increased from 5.6 × 10^14^ cm^−3^ to 5.6 × 10^17^ cm^−3^ at 850 K. The author claimed that the annealing effect is related to the formation of V_Ga_^—^V_O_^++^ complexes as a shallow acceptor center with *E_a_* = 0.17 eV activation energy.

Device applications require higher hole concentrations (at operating temperature), which could be achieved via external acceptor impurity incorporation.

There are already extensive theoretical studies (standard density functional theory (DFT and DFT with GGA+U) of acceptor impurity doping of *β*-Ga_2_O_3_ in order to identify efficient *p*-type dopant. Kyrtsos et al. [116] demonstrated by DFT calculations that dopants, such as Zn, Li, and Mg, will introduce deep acceptor level with ionization energies of more than 1 eV, thus, they cannot contribute to the *p*-type conductivity. However, this result could be influenced by the underestimation of the bandgap due to the semi-local approach. Varley et al. [117] predicted that self-trapped holes are more favorable than delocalized holes due to their energies and by theoretical calculation (self-trapping energy is 0.53 eV and barrier to trapping is 0.10 eV). This indicates that free holes are unstable and will spontaneously localize towards small polarons.

Lyons [118] examined the elements of group 5 and group 12 (Be, Mg, Ca, Sr, Zn, Cd) as acceptor impurities in *β*-Ga_2_O_3_ by hybrid DFT, all of them will exhibit the acceptor ionization levels of more than 1.3 eV. Mg was determined to be the most stable acceptor species, followed by Be. Sun et al. [119] used ab initio calculations to simulate the doping by Ge, Sn, Si, N, and Cl. Among them, N has been predicted to be a deep acceptor with an impurity level of 1.45 eV, as it has a similar atomic size as oxygen but has one less valence electron, and a higher *2p* orbital than oxygen. While all others act as donors, another ab initio calculation also demonstrated that nitrogen doping could introduce an acceptor level at 1.33 eV above the VBM.

Very recently, Goyal et al. [120] simulated a growth-annealing-quench sequence for hydrogen-assisted Mg doping in Ga_2_O_3_ by using the first principles defect theory and defect equilibrium calculations. The H_2_O partial pressure and H exposure can strongly influence the Mg dopants concentration during the growth, by increasing the solubility limit of the acceptor, or by reducing the compensation. A conversion from *n*-type to *p*-type was achieved by annealing at O-rich/H-poor conditions. A Fermi level at +1.5 eV above the VB has been found after quenching.

Doping with two elements (co-doping) has been predicted by DFT which showed a promising method to obtain *p*-type *β*-Ga_2_O_3_, as it can break the solubility limit of mono-doping and improves the photoelectric properties of semiconductor materials which results in increasing the conductivity.

The principle is to increase carrier concentration and decrease the compensating defect formation energy. This is inherently caused by the localized nature of the O2 *p*-derived VB that leads to difficulty in introducing shallow acceptors and large hole effective mass [121].

Co-doping has been successfully used for II-VI compounds, co-doping containing N (Zn-N, N-P, Al-N, and In-N) has been demonstrated to be an effective way to improve the *p*-type conductivity [122,123,124], in particular, Zhang et al. [124] predicted two shallow impurity levels above the VB of about 0.149 eV and 0.483 eV in N–Zn co-doped *β*-Ga_2_O_3_. Co-doping by N-P made an acceptor level decrease ~0.8 eV, and an impurity level appears at 0.55 eV above the VB of *β*-Ga_2_O_3_. A significant loss of holes’ effective mass was also evidenced [124]. There are a few experimental works reported regarding *p*-type doping of gallium oxide. Mg-doped *β*-Ga_2_O_3_was studied by Qian et al. [125] for the photo-blind detector, and the *β*-Ga_2_O_3_ containing 4.92 at% Mg has shown an acceptor level by XPS. A variation of bandgap has also been reported [83,126] however, the Hall effect measurement validity failed at room temperature due to the very high resistivity of the samples [127].

Suet al. [128] deposited Mg-Zn co-doped *β*-Ga_2_O_3_ on sapphire (0001), however, antisites’ impurity defects (i.e., ZnGa and GaZn) were determined as deep acceptors (0.79 eV for ZnGa and 1.00 eV for GaZn) by absorption spectra. Feng et al. [129] demonstrated Zn doping (1.3–3.6 at%) in *β*-Ga_2_O_3_nanowires can reduce the bandgap slightly, they also proved the *p*-type conductivity by making *p*-*n* junction. Chikoidze et al. [24] suggested that Zn in *β*-Ga_2_O_3_ has an amphoteric nature: it can be an acceptor as Zn_Ga_ defect and at the same time, a donor being in Zn_i_ interstitial sites. It was shown that in (0.5%) Zn:Ga_2_O_3_ the auto-compensation of donor (Zn_i_) -acceptor (Zn_Ga_) defects takes place. 

Islam et al. [130] reported that hydrogen annealing could vastly reduce the resistivity and reach a remarkable hole density of ~ 10^15^ cm^−3^ at room temperature. Besides, the ionization energy of acceptor is as low as 42 meV by incorporation of hydrogen in the lattice. This improvement is related to hydrogen decorated gallium vacancies V_Ga-H_: during the diffusion of hydrogen into the Ga_2_O_3_crystal, H^+^ absorbed at the surface will be attracted toward the V_Ga_^3−^, it stabilizes the negative charge and thus lowers the acceptor level. This mechanism leads to H^+^ decorated Ga-vacancy V_Ga-2H_^1−^ and, therefore, the *p*-type conductivity.

Nitrogen-doped *p*-Ga_2_O_3_ has been experimentally achieved by non-conventional growth technique. Wu et al. [131] demonstrated a multi-step structural phase transition growth from hexagonal P6_3_mc GaN to rhombohedral R3C α-GaN_x_O_3(1-x)/2_ and realized the monolithic C2/m N-doped *β*-Ga_2_O_3_ thin layer finally with an acceptor ionization energy of 0.165 eV. The resistivity, hole concentration, and hole mobility are 17.0 Ω.cm, 1.56 × 10^16^cm^−3^, and 23.6 cm^2^V^−1^s^−1^, respectively, by employing the Hall effect measurement. A performant field-effect transistor was also fabricated based on this *p*-type *β*-Ga_2_O_3_. Clearly, further experimental studies of optimal acceptor defects with room temperature activation are required.

### 3.4. Gallium Oxide Power Rectifiers

**Figure 5:**
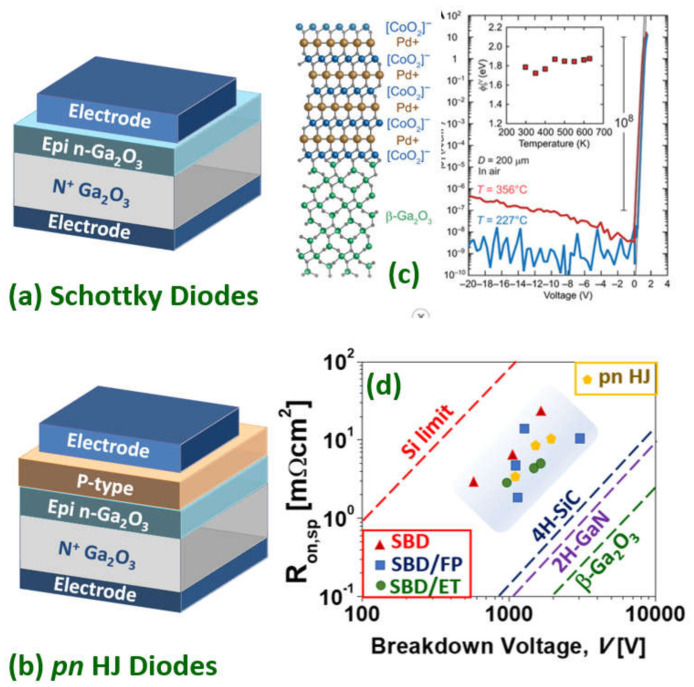
Schematics of (**a**) vertical Ga_2_O_3_ Schottky diodes and (**b**) *p*-*n* heterojunction diodes. (**c**) A PdCoC_2_/Ga_2_O_3_ exhibiting the ultra-large Schottky barrier of 1.8 eV. (**d**) Baliga’s FOM for selected Schottky and p-n HJ diodes from the literature. Panel (**c**) adapted with permission from Harada et al. [137] © 2022 AAAS 4.0 (CC BY-NC). Adapted with permission from [12] © 2021 copyright Society of Photo-Optical Instrumentation Engineers (SPIE).

Once the device-grade epitaxial layers have been grown either homo- (bulk Ga_2_O_3_) hetero- (e.g., sapphire, silicon), or both, the simplest electronic devices one can define are rectifiers. In a Schottky rectifier, the counter-electrode (cathode) is processed to allow low resistance Ohmic contact while the anode contact is intended as a Schottky junction over a lightly doped epitaxy; it conducts electrons in the forward mode while sustaining large electric fields (by the creation of a depletion space charge region) in the reverse mode. As mentioned previously, devices using Ga_2_O_3_ are primarily limited to unipolar devices and Schottky diodes are made, in general, on *n*-type semiconductor layers as electrons are lighter than holes. However, it is also important to consider the appropriate metal contacts to Ga_2_O_3_ as they are responsible for connecting the semiconductor to the surrounding electrical circuit/system and parameters such as the Schottky barrier height are crucial. For different contacts to Ga_2_O_3_, such as in GaN and AlGaN, which utilize stacks of different metals [132], this decision can make an important difference to the nature of the contact. Regarding Schottky contacts to Ga_2_O_3_,Ni/Au is a common choice (see Table 2). Other Schottky contacts investigated include Pt, Ni, Cu, W, Ir, TiN/Au, Pt/Ti/Au, Ni/Au, ndPt/Au [133,134,135,136]. Very recently, an ultra-large Schottky barrier of ~1.8 eV was extracted for all-oxide PdCoO_2_/*β*-Ga_2_O_3_ Schottky diodes [137]. The polar layered structure of PdCoO_2_ generates electric dipoles, realizing a large Schottky barrier height of ~1.8 eV (well beyond the 0.7 eV expected from the basal Schottky–Mott relation) along with a large on/off ratio approaching 10^8^, even at a high temperature of 350 °C (Figure 5c). As there are a number of polar oxides, this is a promising approach to increase the reverse blocking voltage of Ga_2_O_3_ diodes [138].

In the counter-electrode, highly doped regions beneath the metallization are deployed to assist ohmicity of the contacts [139]. The dopants for this have previously been discussed. Another approach to this is using thin films of highly-conducting oxides [140].

Ohmic contacts to *β*-Ga_2_O_3_ are commonly based on Ti/Au, however other metal contacts have been utilized, such as In, Ti, Ti/Al/Au, In/Au, and Ti/Al/Ni/Au. Besides, there are other metals which have exhibited pseudo Ohmic behavior including Zr, Ag, and Sn [132]. This pseudo nature meant that, initially, ohmicity was observed but, after annealing, rectifying behavior became dominant. Therefore, the Schottky/Ohmic nature is also dependent upon the Ga_2_O_3_′s surface/interface states together with the exact choice of metal stack, explaining, in turn, the varying contact resistivity of certain metals. While delivering low contact resistance, it is worth mentioning that Au is not considered a CMOS-compatible metal. This is an issue shared with GaN-based technology [148].

For the continued development of high voltage *β*-Ga_2_O_3_devices, edge termination is an important aspect as it is with its Si, GaN, and 4H-SiCcounterparts. Edge termination in *β*-Ga_2_O_3_is being explored and focused specifically on field plates (FP), imparted edge termination (ET), guard ring field plates, thermally oxidized termination, beveled mesas, and trench. These techniques are all deployed to further manage the electrical field to reduce the electric field crowding at the diode edges to increase its blocking capabilities. SBD devices can be made with either a vertical architecture, using homoepitaxial Ga_2_O_3_ or with a lateral architecture using either homo- or heteroepitaxial (e.g., on sapphire) Ga_2_O_3_. In general, the vertical structure is preferred as the device pitch is reduced and the encapsulation is simpler. Hu et al. [141] demonstrated a field-plated lateral β-Ga_2_O_3_ SBD on a sapphire substrate with a reverse blocking voltage of more than 3 kV, an *R_on_* of 24.3 mΩcm^2^ (anode–cathode spacing 24 μm), and an FOM >0.37 GWcm^−2^ (while an FOM of ~500 GWcm^−2^ was achieved as the anode-cathode spacing (and *V_br_*) was reduced). Zhou et al. [149] implemented a Mg implanted ET device on a vertical β-Ga_2_O_3_ SBD with a reverse blocking voltage of 1.55 kV and a low specific on-resistance of 5.1 mΩcm^2^ (epi thickness 10 μm) and an FOM of 0.47 GWcm^−2^. Analogously, Lin et al. [150] implemented a guard ring with or without an FP on vertical SBDs. The terminated devices exhibited a specific on-resistance of 4.7 mΩcm^2^ and a V_br_ of 1.43 kV. Wang et al. [151] implemented a thermally oxidized termination on a vertical SBD with a V_br_ of 940 V, a specific on-resistance of 3.0 mΩcm^2^, and an FOM of 0.295 GWcm^−2^. Allen et al. [152] implemented a small-angle beveled field plate (SABFP), on thinned Ga_2_O_3_ substrates and a non-punch-through vertical SBD design rendering a V_br_ of 1100 V, a peak electric field of 3.5 MVcm^−1^, and an FOM of 0.6 GWcm^−2^.

Somehow the state of the art is given by Li et al. [153]. They demonstrated an FP vertical Ga_2_O_3_ trench SBDs with a V_br_ of 2.89 kV (which is ~500 V higher than those without FPs). The trench SBDs exhibited a differential specific on-resistance of 10.5 (8.8) mΩcm^2^ from DC (pulsed) measurements leading to an FOM of 0.80 (0.95) GWcm^−2^. This Baliga’s power FOM is approaching that for the best vertical SBD GaN devices (e.g., 1.7 GWcm^−2^ [154]) but is still several times smaller than lateral AlGaN/GaN SBD (e.g., 3.6 GWcm^−2^ [155]) and bipolar *p*-*n* vertical GaN diodes (e.g., ~4.6 GWcm^−2^ [156]). Both, the 2D gas formed at the AlGaN/GaN interface and the bipolar injection are effective ways of further reducing the on-resistance in these devices while keeping the breakdown voltage high. The lack of low resistivity *p*-type layer for the anode has to date, prevented a competitive homojunction p-n Ga_2_O_3_ diode, but *p*-*n* heterojunction diodes have been realized by integrating *n*-type Ga_2_O_3_ with *p*-type semiconductors, such as CuO (1.49 kV) [157] and NiO (1.06 kV/1.86kV) [158,159].Nickel oxide as the *p*-type blocking layer in heterojunction power diodes resulted in a particularly promising approach with this NiO/Ga_2_O_3_device [160] yielding a Baliga’s FOM of 0.33 GWcm^−2^ (Figure 5c,d).

Recently, extremely high-*k* dielectrics have been explored for electric field management in WBG semiconductor-based lateral and vertical device structures [160,161,162,163,164]. According to the TCAD simulations of Roy et al. [165], a super-dielectric Ga_2_O_3_ SBD with practically achievable device dimensions with extremely high FOM should be possible; e.g., 20kVcanbeachievedforan *R_on_* of 10 mΩ-cm^2^ with a dielectric constant of 300, a Ga_2_O_3_ width/dielectric width ratio of 0.2, and an aspect ratio (drift layer length (anode to cathode spacing)/drift layer width ratio) of 10 resulting in a PFOM of 40 GWcm^−2^ (surpassing the theoretical unipolar FOM of *β*-Ga_2_O_3_SBD by four times).

### 3.5. Gallium Oxide Power Transistors

A power MOSFET fabrication process generally includes a number of technological steps including either gate dielectrics, surface passivation, drain/source ohmic contacts, implant doping, isolation, mesa etch, or in combination. Due to the large bandgap of Ga_2_O_3_, the most suitable gate insulators are those with enough (conduction and valence) band-offsets to avoid current injection through the gate (e.g., SiO_2_ and Al_2_O_3_ and perhaps other oxides such as Y_2_O_3_, MgO, and Mg_2_AlO_4_). While balancing the dielectric constant to achieve more gate capacitance and more carriers in the conductive channel [166]. Defining a contact region by implantation, such as in Si, SiC, and GaN power MOSFET technologies, is a usual choice [167], in Ga_2_O_3_ this is typically n^+^ Si-ion implantation. While other techniques have been suggested to further decrease the contact resistivity, such as formation of surface states [168] or the adoption of a TCO as a metallic interface [169].

As in, the more mature, AlGaN/GaN HEMT technology, Ohmic contacts are typically made with a multilayer metal stack consisting of an adhesion layer (e.g., Ti, Ta), an overlayer (Al), a barrier layer (e.g., Ni, Ti, Mo), and a capping of Au [170,171]. Nevertheless, it has been argued that simpler metal structures, such as Ti/Ga_2_O_3_, are also efficient if there is an oxygen deficient Ga_2_O_3_ surface [172] (a double charged oxygen vacancy is a well-known intrinsic donor in oxides [107]). Indeed, Yao et al. [132] suggested that the surface states appear to have a more dominant role in the transformation from a Schottky to an Ohmic interface than the choice of metal.

**Figure 6:**
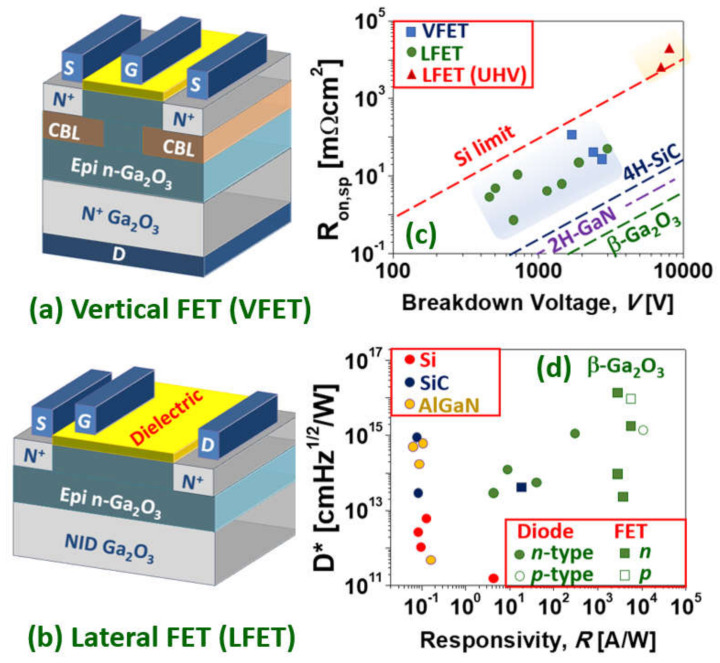
Schematics of (**a**) a vertical Ga_2_O_3_ power transistor (VFET) and (**b**) a lateral transistor (LFET). (**c**) Baliga’s FOM for selected LFETs and VFETs from the literature. (**d**) Prospects of Ga_2_O_3_ devices as UV PDs, D* refers to specific detectivity; dots symbols referrer to diodes (either SBD or MSM), while square symbols denote transistors (data adapted from Wu et al. [131]). Adapted with permission from [12] © 2021 copyright Society of Photo-Optical Instrumentation Engineers (SPIE).

As with power SBDs, power MOSFETs can be defined in a vertical Ga_2_O_3_ homoepitaxial structure (typical of SiC power MOSFETs) and lateral structure (typical of AlGaN/GaN power HEMTs) which can be either homoepitaxial or heteroepitaxial (Figure 6). Ga_2_O_3_ power MOSFETs are mostly unipolar *n*-type and operate in depletion mode (D-mode or normally-on) but a number of techniques have been reported to make enhancement mode (E-mode or normally-off) Ga_2_O_3_ devices. For example, Chabak et al. [173] reported an enhancement-mode *β*-Ga_2_O_3_ MOSFETs on a Si-doped homoepitaxial channel grown by molecular beam epitaxy and, using a gate recess process to partially remove the epitaxial channel under the 1-μm gated region to fully deplete at zero gate bias. With a breakdown voltage of 505 V (8 mm source-drain spacing), a maximum current density of 40 mA mm^−1^, and an on/off ratio of 10^9^. Hu et al. [174] achieved (in 2018) a larger blocking voltage (1.075 kV), a larger threshold voltage (1.2–2.2 V), and a larger output current (~500 A cm^−2^) in a first demonstration of vertical E-mode MOSFET with significatively larger FOM (~80 MW cm^−2^).

The E-mode was accomplished by doping profiling in a FinFET design (a type of 3D, non-planar transistor which has become the usual layout for the smallest CMOS 14 nm, 10 nm, and 7 nm nodes). This kind of E-mode vertical power device was later optimized to sustain up to a blocking voltage of 1.6kV [175], a threshold voltage of 2.66 kV, a maximum current density of 25.2 mWcm^2^, and a record FOM of 280 MW cm^−2^ [176]. Among D-mode devices, the ones reported by Lv et al. [177] stand out for exhibiting a particularly large FOM. They reported (in 2019) [177] source-FP *β*-Ga_2_O_3_ MOSFETs on a Si-doped/Fe-doped semi-insulating *β*-Ga_2_O_3_ substrate exhibiting 222 mA mm^−1^ (18 mm source-drain spacing) with on-resistance of 11.7 mΩcm^2^, a V_br_ of 680 V and an FOM of 50.4 MWcm^−2^. Later (in 2020) [178], they adopted a T-shaped gate and source connected FP structure to increase the V_br_ up to 1.4 kV/2.9 kV (for 4.8 μm/17.8 μm source-drain spacing), with a specific on-resistances of 7.08 mΩcm^2^/46.2 mΩcm^2^. These yielded a record high FOM of 277 MW cm^−2^, together with negligible gate or drain pulsed current collapse and a drain current on/off ratio of 10^9^.

Other lateral D-mode devices with high FOM were reported by Tetzner et al. [179]. By using sub-μm gate lengths (combined with gate recess) and optimization of compensation-doped high-quality crystals, implantation based inter-device isolation, and SiNx-passivation, breakdown voltages of 1.8 kV and an FOM of 155 MW cm^−2^ were achieved. In 2020, Sharma et al. [180] reported Ga_2_O_3_ lateral D-mode field-plated MOSFETs exhibiting an ultra-high V_br_ of 8.03 kV (70 mm) by using polymer SU8 passivation. The current was rather low, however, due to plasma-induced damage of channel and access regions resulting in an impractical FOM of 7.73 kW cm^−2^ (i.e., not above the silicon limit). As reported by Kalarickal et al. [164], ultra-high-*k* ferroelectric dielectrics, such as BaTiO_3_, can, in principle, provide an efficient field management strategy by improving the uniformity of electric field profile in the gate-drain region of lateral FETs. High average breakdown fields of 1.5 MV/cm (918 V) and 4 MVcm^−1^ (201 V) were demonstrated for gate-drain spacings of 6μm and 0.6 μm, respectively, in 𝛽-Ga_2_O_3_, at a high channel sheet charge density of 1.8×10^13^ cm^−2^. An elevated sheet charge density together with a high breakdown field enabled a record power FOM of 376 MWcm^−2^ at a gate-drain spacing of 3 μm (Figure 6c). As in the case of SBDs, these performances for the Ga_2_O_3_ devices are already impressive and well beyond the silicon limit but still lag behind the best (much more mature) GaN devices in their respective power ratings [181,182].

All the above power MOSFET devices are unipolar *n*-type. These devices are sometimes referred as MISFETs so as to distinguish them from the conventional p-n junction based MOSFETs, since there are no *p*-regions in these MISFETs [175]. As mentioned in the previous sections, there are, however, several reports of *p*-type Ga_2_O_3_in nominally undoped, H-doped and N-doped *β*-Ga_2_O_3_. In particular, Wuetal. [131] proposed a growth mechanism of multistep structural phase transitions from hexagonal P63mc GaN to rhombohedral R3c *α*-GaN_x_O_3(1−x)/2_,and finally to monolithic C2/m N-doped *β*-Ga_2_O_3_. This improves the crystalline quality, facilitates acceptor doping, increases the acceptor activation efficiency, and thus enhances the *p*-type conductivity (acceptor ionization energy of 0.165 eV, Hall resistivity of 17.0 Ωcm, Hall hole mobility of 23.6 cm^2^V^−1^s^−1^, hole concentration of 1.56×10^16^ cm^−3^). P-type *β*-Ga_2_O_3_ films-based lateral MOSFET deep-ultraviolet (DUV) PDs were fabricated with extremely high responsivity (5.1×10^3^ A/W) and detectivity (1.0×10^16^Jones) under 250 nm light illumination (40 μW/cm^2^) conditions. Figure 6d shows the responsivity and detectivity (D*) for state-of-the-art DUV PDs based on various WBG materials (adapted from [131]), in which it can be seen how *β*-Ga_2_O_3_ surpasses conventional Si-, SiC-, and AlGaN-based devices in terms of responsivity and detectivity.

## 4. Other Emerging Oxide Semiconductors for Power Electronics

Ga_2_O_3_phase engineering: Owing to the nonpolar nature of β-Ga_2_O_3_ crystals, modulation-doped heterostructure is one of the possible approaches to realize Ga_2_O_3_-based FETs [183]. Analogously, *p*-type semiconductors (e.g., *p*-type nitrides such as GaN) may be introduced to yield normally-off β-Ga_2_O_3_ field-effect transistors with tunable positive threshold voltages [184]. Other phases of Ga_2_O_3_ have also received attention due to potentially favorable growth characteristics, and to the possibility of polarization engineering made possible by the polar nature of their crystal structures. In principle, this polarization could be utilized to produce Ga_2_O_3_ two-dimensional electron gases (2DEGs) in analogy with GaN/AlN-based transistors [185].

Ga_2_O_3_alloy engineering: The aluminum gallium oxide, Al_x_Ga_1-x_O_3_, is a ternary alloy of Al_2_O_3_ and Ga_2_O_3_. It was already noted by Roy [186] in 1952 that the gallium ion closely resembles the aluminum ion and substitutes for it in several structures. Because *β*-(AlGa)_2_O_3_ is not the energetically favored crystalline phase for large Al compositions, the crystal converts to competing structural phases when grown on β-Ga_2_O_3_ substrates [187]. Thus, it has been difficult to obtain gallium oxide UWBG materials exceeding the bandgap of ~6 eV which is available to the materials in the nitride family in AlN. Very recently however, it was found that single-crystalline layers of α-(AlGa)_2_O_3_ alloys spanning bandgaps of 5.4–8.6 eV can be grown by molecular beam epitaxy [188]. By varying the alloy composition, bandgap energies from ~5.4 up to 8.6 eV with a bowing parameter of 1.1 eV are achieved, making α-(Al_x_Ga_1−x_)_2_O_3_ the largest bandgap epitaxial material family to date. If these layers can be controllably doped, it would pave the way for α-(Al_x_Ga_1−x_)_2_O_3_–based high-power heterostructure electronic and photonic devices at bandgaps far beyond all materials available today [189].

Spinel electronics: The spinel zinc gallate, ZnGa_2_O_4_, is a nearly stoichiometric mixed oxide made of Ga_2_O_3_ and ZnO.A potential advantage of spinel ZnGa_2_O_4_ is its great dopability prospects owing to the spinel‘s inherent diversity in cation coordination possibilities [106]. Normal spinels have all A cations in the tetrahedral site and all B cations in the octahedral site, e.g., Zn-tetrahedral site Zn^2+^(T_d_) and Ga-octahedral site Ga^3+^(O_h_), so that normal ZnGa_2_O_4_ is Zn(^2+^[T_d_])Ga_2_(^3+^[O_h_])O_4_(^2−^). The spinel’s off-stoichiometry, from the ideal 1:2:4 proportions, or the creation of cation antisite defects are known routes for doping these compounds. Dominant defects in spinels are antisite donors (e.g., Zn_Ga_) or donor-like Ga^3+^(O_h_)-on-T_d_ and antisite acceptors (e.g., GaZn) with acceptor-like Zn^2+^(T_d_)-on-O_h_ antisite defects resulting in an intrinsic bipolar power semiconductor [190]. ZnGa_2_O_4_ is therefore a potential outstanding UWBG (~5 eV) oxide semiconductor but is only one among the many possible spinel oxides. There are over 1000 compounds that are known to crystalize in the spinel structure. The sub-family of spinel oxides is a large and important class of multi-functional oxide semiconductors with many optoelectronics applications in areas such as batteries, fuel cells, catalysis, photonics (phosphors, bio-imaging, photodetectors), spintronics (magnets, bio-magnets), or thermoelectricity [191]. Other magnesium-based Ga-spinels, such as MgGa_2_O_4_and Zn_1-x_Mg_x_Ga_2_O_4_, are related oxides that are currently being investigated [192,193].

## 5. Conclusions

The rational use of electrical energy and information are central themes in the greatest climatic challenge of the 21st century. UWBG oxides, such as Ga_2_O_3_ and related materials, are promising power electronic candidates since their critical electric field is large compared to beyond silicon WBG (i.e., SiC and GaN), while still yielding a moderate mobility, high quality epi-layers, and large bulk single crystals (more than 6-inch) using low cost and scalable fabrication approaches. During the last decade, the Ga_2_O_3_ power diode and transistor progress has been impressive, with devices now approaching the frontier of the field. The material system also opens new optoelectronics avenues (owing its UVC spanning bandgap), and new electronics perspectives based on stabile interfaces and a natural integration with extremely high-*k* functional oxides. The advances offered by Ga_2_O_3_ are also opening the door to many more UWBG oxides (the largest family of wide bandgap semiconductors), such as the spinel, ZnGa_2_O_4_, along with many more that are anticipated. Therefore, the ever-increasing family of UWBG oxides is at the very frontier of a more efficient energy electronics which is adapted to tackle the 21st century climatic targets, although there still is a lot of room for performance improvements, technical innovation, and new discoveries.

## Figures and Tables

**Table 1:** Overview of *β*-Ga_2_O_3_ bulk crystal growth methods.

Method	Verneuil	FZ	CZ	EFG	VB
Schematic illustration	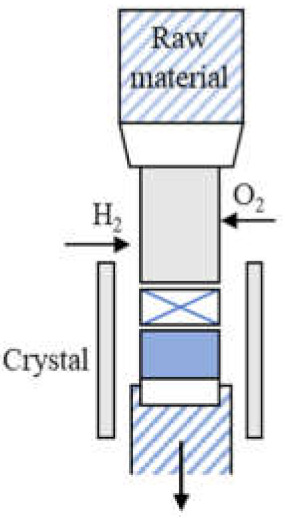	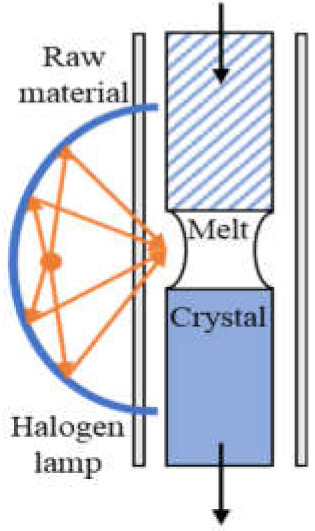	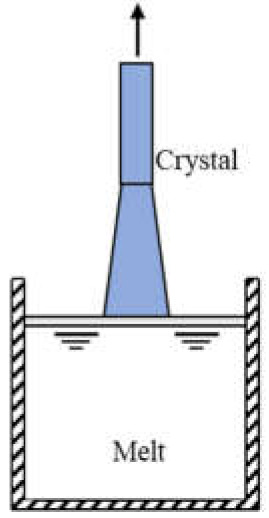	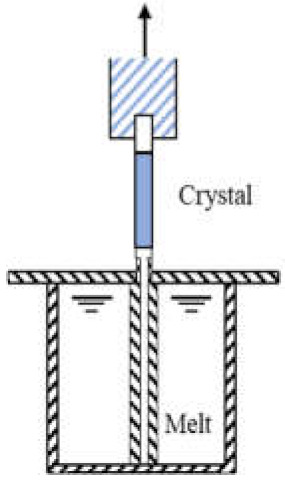	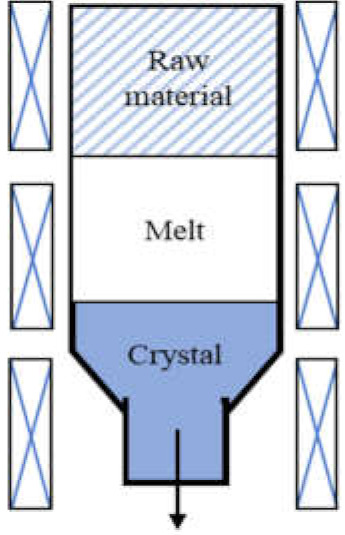
Bulk size	3/8-inch diameter 1-inch length	1-inch diameter	2-inch diameter	6-inch width 4-inch diameter	2-inch diameter
Growth rate (mm/h)	10	20–40	2	15	5
FWMH	-	22 arcsec	22–50 arcsec	17 arcsec	10–50 arcsec
Dislocation density	-	-	~10^3^ cm^−2^	10^3^ cm^−2^	10^2^–2 ×10^3^ cm^−2^
Residual impurity	2 × 10^18^cm^−3^	~10^17^ cm^−3^ (Si, Sn)	~10^16^ cm^−3^ (Si)	~10^17^ cm^−3^ (Si)	~several tens wt.ppm (Rh)
Intentional doping	~10^19^ cm^−3^ (Mg), ~10^21^ cm^−3^ (Zr) at 900 °C	~10^19^ cm^−3^ (Nb, Ta)	~10^19^ cm^−3^ (Sn, Si, Hf)	6-7 × 10^18^ cm^−3^ (Si, Sn)	3.6 × 10^18^ cm^−3^ (Sn)
Refs.	[21,28,37,38]	[34,38,39,40,41,42,43]	[16,17,30,33,43,44,45]	[16,17,46,47]	[35,36,48]

**Table 2:** Table displaying varying SBD designs: L—lateral, V—vertical, TCO—thin conductive oxide film, FP—field plate, BET—bevel edge termination, FPET—field plate edge termination, MDS—metal-dielectric-semiconductor Schottky diode. Included here are different structures which exhibited SBD (some exhibiting Schottky contacts as opposed to useable device) using a range of different designs and metal stacks.

Device Configuration	Schottky Metal Stack	Ohmic Metal Stack	$V_{br}$	Ideality Factor	Ref.
V-SBD-BET	Ni/Au	Ti/Al/Ni/Au	427 V	1.07	[133]
V-SBD-FP	Ni/Au	Ti/Au	730 V V	1.02	[139]
V-SBD-FPET	Ni/Au	Ti/Au	1722 V	1.03	[140]
L-SBD-FP	Ni/Au	Ti/Au	<3 kV	~1.25	[141]
L-SBD	Ni/Au	Ti/Au	1.7 kV	-	[142]
L-SBD	Pt	Ti/Au	-	1.40	[134]
L-SBD	Ir	Ti/Au	-	1.45	[134]
V-SBD	Ni	Ti/Au	-	1.57	[134]
L-SBD	Ni	Ti/Au	-	1.33	[134]
V-SBD	Cu	Ti/Au	-	1.53	[134]
L-SBD	W	Ti/Au	-	1.4	[134]
V-SBD	Ni/Au	Sn	~210 V	3.38	[143]
L-SBD	Ptx	Ti/Al/Au	-	-	[144]
V-SBD	Pt/Au	Ti/Au	-	-	[135]
V-SBD	TiN	Ti/Au	-	1.03	[145]
V-SBD	Pt/Ti/Au	Ti/Au	-	1.03	[136]
V-SBD-TCO	SnO/Ti	Ti/Au	-	1.09	[146]
V-MDS(TiO_2_)	Ni/Au	Ti/Au	1010 V	-	[147]

## Data Availability

Not applicable.

## References

[1] IPCC Working Group I Report, Climate Change 2021: The Physical Science Basis. www.ipcc.ch.

[2] US Environmental Protection Agency (EPA) (2021). Greenhouse Gas Emissions: Global Greenhouse Gas Emissions Data. https://www.epa.gov/ghgemissions/global-greenhouse-gas-emissions-data.

[3] Leo Lorenz, Power Device Development Trends—From Silicon to Wide Bandgap?. www.power-and-beyond.com.

[4] Pearton S.J., Ren F., Tadjer M., Kim J. (2018). Perspective: Ga_2_O_3_ for ultra-high power rectifiers and MOSFETS. J. Appl. Phys..

[5] Reese S.B., Remo T., Green J., Zakutayev A. (2019). How Much Will Gallium Oxide Power Electronics Cost?. Joule.

[6] Chu S., Cui Y., Liu N. (2017). The path towards sustainable energy. Nat. Mater..

[7] Millan J., Godignon P., Perpiñà X., Perez-Tomas A., Rebollo J. (2013). A Survey of Wide Bandgap Power Semiconductor Devices. IEEE Trans. Power Electron..

[8] Spaziani L., Lu L. Silicon, GaN and SiC: There’s room for all: An application space overview of device considerations. Proceedings of the 2018 IEEE 30th International Symposium on Power Semiconductor Devices and ICs (ISPSD).

[9] Jones E.A., Wang F.F., Costinett D. (2016). Review of Commercial GaN Power Devices and GaN-Based Converter Design Challenges. IEEE J. Emerg. Sel. Top. Power Electron..

[10] Tsao J.Y., Chowdhury S., Hollis M.A., Jena D., Johnson N.M., Jones K.A., Kaplar R.J., Rajan S., Van de Walle C.G., Bellotti E. (2017). Ultrawide-Bandgap Semiconductors: Research Opportunities and Challenges. Adv. Electron. Mater..

[11] Perez-Tomas A., Teherani F.H., Bove P., Sandana E.V., Chikoidze E., Jennings M.R., Rogers D.J., Russell S.A.O. Wide and ultra-wide bandgap oxides: Where paradigm-shift photovoltaics meets transparent power electronics. Proceedings of the SPIE 10533, Oxide-Based Materials and Devices IX.

[12] Perez-Tomas A., Chikoidze E., Rogers D.J. A walk on the frontier of energy electronics with power ultra-wide bandgap oxides and ultra-thin neuromorphic 2D materials. Proceedings of the SPIE 11687, Oxide-Based Materials and Devices XII.

[13] Perez-Tomas A., Lodzinski M., Guy O.J., Jennings M.R., Placidi M., Llobet J., Gammon P.M., Davis M.C., Covington J.A., Burrows S.E. (2009). Si/SiC bonded wafer: A route to carbon free SiO_2_ on SiC. Appl. Phys. Lett..

[14] Nawaz M. (2019). Introductory Chapter: Earth Crust-Origin, Structure, Composition and Evolution. Earth Crust.

[15] Jackson I. (1998). The Earth’s Mantle—Composition, Structure and Evolution.

[16] Higashiwaki M., Sasaki K., Murakami H., Kumagai Y., Koukitu A., Kuramata A., Masui T., Yamakoshi S. (2016). Recent progress in Ga_2_O_3_ power devices. Semicond. Sci. Technol..

[17] Kuramata A., Koshi K., Watanabe S., Yamaoka Y., Masui T., Yamakoshi S. Bulk crystal growth of Ga_2_O_3_. Proceedings of the SPIE 10533, Oxide-Based Materials and Devices IX.

[18] Playford H.Y., Hannon A.C., Barney E.R., Walton R.I. (2013). Structures of Uncharacterised Polymorphs of Gallium Oxide from Total Neutron Diffraction. Chem. A Eur. J..

[19] Geller S. (1960). Crystal Structure of β—Ga_2_O_3_. J. Chem. Phys..

[20] Blasse G., Bril A. (1970). Some observations on the luminescence of β-Ga_2_O_3_. J. Phys. Chem. Solids.

[21] Lorenz M., Woods J., Gambino R. (1967). Some electrical properties of the semiconductor β Ga_2_O_3_. J. Phys. Chem. Solids.

[22] Tippins H.H. (1965). Optical Absorption and Photoconductivity in the Band Edge of β−Ga_2_O_3_. Phys. Rev..

[23] Nouketcha F.L.L., Cui Y., Lelis A., Green R., Darmody C., Schuster J., Goldsman N. (2020). Investigation of Wide- and Ultrawide-Bandgap Semiconductors From Impact-Ionization Coefficients. IEEE Trans. Electron. Devices.

[24] Chikoidze E., Tchelidze T., Sartel C., Chi Z., Kabouche R., Madaci I., Rubio C., Mohamed H., Sallet V., Medjdoub F. (2020). Ultra-high critical electric field of 13.2 MV/cm for Zn-doped p-type β-Ga_2_O_3_. Mater. Today Phys..

[25] Baliga B. (1989). Power semiconductor device figure of merit for high-frequency applications. IEEE Electron Device Lett..

[26] Ma N., Tanen N., Verma A., Guo Z., Luo T., Xing H., Jena D. (2016). Intrinsicelectronmobilitylimits in β-Ga_2_O_3_. Appl. Phys. Lett..

[27] Fu B., Jia Z., Mu W., Yin Y., Zhang J., Tao X. (2019). A review of β-Ga_2_O_3_ single crystal defects, their effects on device performance and their formation mechanism. J. Semicond..

[28] Stuchlikova T.H., Stuchlik J., Remes Z., Taylor A., Mortet V., Ashcheulov P., Gregora I., Krivyakin G., Volodin V. (2020). High-Temperature PIN Diodes Based on Amorphous Hydrogenated Silicon-Carbon Alloys and Boron-Doped Diamond Thin Films. Phys. Status Solidi.

[29] Chase A.O. (1964). Growth of beta-Ga_2_, O_3_ by the Verneuil Technique. J. Am. Ceram. Soc..

[30] Galazka Z., Ganschow S., Irmscher K., Klimm D., Albrecht M., Schewski R., Pietsch M., Schulz T., Dittmar A., Kwasniewski A. (2020). Bulk single crystals of β-Ga_2_O_3_ and Ga-based spinels as ultra-wide bandgap transparent semiconducting oxides. Prog. Cryst. Growth Charact. Mater..

[31] Galazka Z., Irmscher K., Schewski R., Hanke I.M., Pietsch M., Ganschow S., Klimm D., Dittmar A., Fiedler A., Schroeder T. (2019). Czochralski-grown bulk β-Ga_2_O_3_ single crystals doped with mono-, di-, tri-, and tetravalent ions. J. Cryst. Growth.

[32] Galazka Z., Schewski R., Irmscher K., Drozdowski W., Witkowski M.E., Makowski M., Wojtowicz A.J., Hanke I.M., Pietsch M., Schulz T. (2019). Bulk β-Ga_2_O_3_ single crystals doped with Ce, Ce+Si, Ce+Al, and Ce+Al+Si for detection of nuclear radiation. J. Alloys Compd..

[33] Irmscher K., Galazka Z., Pietsch M., Uecker R., Fornari R. (2011). Electrical properties of β-Ga_2_O_3_ single crystals grown by the Czochralski method. J. Appl. Phys..

[34] Víllora E.G., Shimamura K., Yoshikawa Y., Aoki K., Ichinose N. (2004). Large-size β-Ga_2_O_3_ single crystals and wafers. J. Cryst. Growth.

[35] Hoshikawa K., Kobayashi T., Matsuki Y., Ohba E. (2020). 2-inch diameter (1 0 0) β-Ga_2_O_3_ crystal growth by the vertical Bridgman technique in a resistance heating furnace in ambient air. J. Cryst. Growth.

[36] Hoshikawa K., Ohba E., Kobayashi T., Yanagisawa J., Miyagawa C., Nakamura Y. (2016). Growth of β-Ga_2_O_3_ single crystals using vertical Bridgman method in ambient air. J. Cryst. Growth.

[37] Harwig T., Schoonman J. (1978). Electrical properties of β-Ga_2_O_3_ single crystals. II. J. Solid State Chem..

[38] Cui H., Mohamed H., Xia C., Sai Q., Zhou W., Qi H., Zhao J., Si J., Ji X. (2019). Tuning electrical conductivity of β-Ga_2_O_3_ single crystals by Ta doping. J. Alloys Compd..

[39] Hossain E., Kulkarni R., Mondal R., Guddolian S., Rahman A.A., Thamizhavel A., Bhattacharya A. (2019). Optimization of Gas Ambient for High Quality β-Ga_2_O_3_ Single Crystals Grown by the Optical Floating Zone Technique. ECS J. Solid State Sci. Technol..

[40] Suzuki N., Ohira S., Tanaka M., Sugawara T., Nakajima K., Shishido T. (2007). Fabrication and characterization of transparent conductive Sn-doped β-Ga_2_O_3_ single crystal. Phys. Status Solidi.

[41] Tomioka Y., Ozaki Y., Inaba H., Ito T. (2019). Compensation effects between impurity cations in single crystals of a wide gap semiconductor β-Ga_2_O_3_ prepared by the floating zone method. Jpn. J. Appl. Phys..

[42] Zhou W., Xia C., Sai Q., Zhang H. (2017). Controlling n-type conductivity of β-Ga_2_O_3_ by Nb doping. Appl. Phys. Lett..

[43] Galazka Z. (2018). β-Ga_2_O_3_ for wide-bandgap electronics and optoelectronics. Semicond. Sci. Technol..

[44] Galazka Z., Uecker R., Klimm D., Irmscher K., Naumann M., Pietsch M., Kwasniewski A., Bertram R., Ganschow S., Bickermann M. (2016). Scaling-Up of Bulk β-Ga_2_O_3_Single Crystals by the Czochralski Method. ECS J. Solid State Sci. Technol..

[45] Saleh M., Varley J.B., Jesenovec J., Bhattacharyya A., Krishnamoorthy S., Swain S., Lynn K.G. (2020). Degenerate doping in β-Ga_2_O_3_ single crystals through Hf-doping. Semicond. Sci. Technol..

[46] Kuramata A., Koshi K., Watanabe S., Yamaoka Y., Masui T., Yamakoshi S. (2016). High-quality β-Ga_2_O_3_single crystals grown by edge-defined film-fed growth. Jpn. J. Appl. Phys..

[47] Yao Y., Sugawara Y., Ishikawa Y. (2020). Identification of Burgers vectors of dislocations in monoclinic β-Ga_2_O_3_ via synchrotron X-ray topography. J. Appl. Phys..

[48] Hoshikawa K., Kobayashi T., Ohba E. (2020). 50 mm diameter Sn-doped (0 0 1) β-Ga_2_O_3_ crystal growth using the vertical Bridgeman technique in ambient air. J. Cryst. Growth.

[49] Aubay E., Gourier D. (1993). Magnetic bistability and Overhauser shift of conduction electrons in gallium oxide. Phys. Rev. B.

[50] He N., Tang H., Liu B., Zhu Z., Li Q., Guo C., Gu M., Xu J., Liu J., Xu M. (2018). Ultra-fast scintillation properties of β-Ga_2_O_3_ single crystals grown by Floating Zone method. Nucl. Instrum. Methods Phys. Res. Sect. A Accel. Spectrom. Detect. Assoc. Equip..

[51] Usui Y., Oya T., Okada G., Kawaguchi N., Yanagida T. (2017). Comparative study of scintillation and optical properties of Ga_2_O_3_ doped with ns2 ions. Mater. Res. Bull..

[52] Yanagida T., Kawaguchi N. (2019). Optical and scintillation properties of alkaline earth doped Ga_2_O_3_ single crystals prepared by the floating zone method. Jpn. J. Appl. Phys..

[53] Zhang S., Lian X., Ma Y., Liu W., Zhang Y., Xu Y., Cheng H. (2018). Growth and characterization of 2-inch high quality β-Ga_2_O_3_ single crystals grown by EFG method. J. Semicond..

[54] Oshima T., Hashiguchi A., Moribayashi T., Koshi K., Sasaki K., Kuramata A., Ueda O., Oishi T., Kasu M. (2017). Electrical properties of Schottky barrier diodes fabricated on (001) β-Ga_2_O_3_substrates with crystal defects. Jpn. J. Appl. Phys..

[55] Oshima Y., Vίllora E.G., Shimamura K. (2015). Quasi-heteroepitaxial growth of β-Ga_2_O_3_ on off-angled sapphire (0 0 0 1) substrates by halide vapor phase epitaxy. J. Cryst. Growth.

[56] Xiong Z.-N., Xiu X.-Q., Li Y.-W., Hua X.-M., Xie Z.-L., Chen P., Liu B., Han P., Zhang R., Zheng Y.-D. (2018). Growth of β -Ga_2_O_3_ Films on Sapphire by Hydride Vapor Phase Epitaxy. Chin. Phys. Lett..

[57] Yao Y., Lyle L.A.M., Rokholt J.A., Okur S., Tompa G.S., Salagaj T., Sbrockey N., Davis R.F., Porter L.M. (2017). (Invited) Growth and Characterization of α-,β-, andε-Ga_2_O_3_ Epitaxial Layers on Sapphire. ECS Trans..

[58] Xiu X., Zhang L., Li Y., Xiong Z., Zhang R., Zheng Y. (2019). Application of halide vapor phase epitaxy for the growth of ultra-wide band gap Ga_2_O_3_. J. Semicond..

[59] Modak S., Chernyak L., Khodorov S., Lubomirsky I., Yang J., Ren F., Pearton S.J. (2019). Impact of Electron Injection and Temperature on Minority Carrier Transport in Alpha-Irradiated ß-Ga_2_O_3_ Schottky Rectifiers. ECS J. Solid State Sci. Technol..

[60] De Santi C., Nardo A., Wong M., Goto K., Kuramata A., Yamakoshi S., Murakami H., Kumagai Y., Higashiwaki M., Meneghesso G. (2019). Stability and degradation of isolation and surface in Ga_2_O_3_ devices. Microelectron. Reliab..

[61] Leach J.H., Udwary K., Rumsey J., Dodson G., Splawn H., Evans K.R. (2019). Halide vapor phase epitaxial growth of β-Ga_2_O_3_ and α-Ga_2_O_3_ films. APL Mater..

[62] Murakami H., Nomura K., Goto K., Sasaki K., Kawara K., Thieu Q.T., Togashi R., Kumagai Y., Higashiwaki M., Kuramata A. (2014). Homoepitaxial growth of β-Ga_2_O_3_layers by halide vapor phase epitaxy. Appl. Phys. Express.

[63] Bin Anooz S., Grüneberg R., Wouters C., Schewski R., Albrecht M., Fiedler A., Irmscher K., Galazka Z., Miller W., Wagner G. (2020). Step flow growth of β-Ga_2_O_3_ thin films on vicinal (100) β-Ga_2_O_3_ substrates grown by MOVPE. Appl. Phys. Lett..

[64] Bin Anooz S., Grüneberg R., Chou T.-S., Fiedler A., Irmscher K., Wouters C., Schewski R., Albrecht M., Galazka Z., Miller W. (2020). Impact of chamber pressure and Si-doping on the surface morphology and electrical properties of homoepitaxial (100) β-Ga_2_O_3_ thin films grown by MOVPE. J. Phys. D Appl. Phys..

[65] Gogova D., Schmidbauer M., Kwasniewski A. (2015). Homo- and heteroepitaxial growth of Sn-doped β-Ga_2_O_3_ layers by MOVPE. CrystEngComm.

[66] Albrecht M., Schewski R., Wouters C., Fielder A., Irmscher K., Galazka Z., Popp A., Anooz S.B., Baldini M., Wagner G. (2021). Ga_2_O_3_ from Materials to Devices. Appl. Phys. Res..

[67] Baldini M., Albrecht M., Fiedler A., Irmscher K., Schewski R., Wagner G. (2016). Editors’ Choice—Si- and Sn-Doped Homoepitaxial β-Ga_2_O_3_ Layers Grown by MOVPE on (010)-Oriented Substrates. ECS J. Solid State Sci. Technol..

[68] Cui R.-R., Zhang J., Luo Z.-J., Guo X., Ding Z., Deng C.-Y. (2021). Microstructure, optical, and photoluminescence properties of β-Ga_2_O_3_ films prepared by pulsed laser deposition under different oxygen partial pressures. Chin. Phys. B.

[69] Leedy K., Chabak K.D., Vasilyev V., Look D.C., Boeckl J.J., Brown J.L., Tetlak S.E., Green A.J., Moser N.A., Crespo A. (2017). Highly conductive homoepitaxial Si-doped Ga_2_O_3_ films on (010) β-Ga_2_O_3_ by pulsed laser deposition. Appl. Phys. Lett..

[70] Shen H., Baskaran K., Yin Y., Tian K., Duan L., Zhao X., Tiwari A. (2019). Effect of thickness on the performance of solar blind photodetectors fabricated using PLD grown β-Ga_2_O_3_ thin films. J. Alloys Compd..

[71] Vu T.K.O., Lee D.U., Kim E.K. (2019). The effect of oxygen partial pressure on band gap modulation of Ga_2_O_3_ grown by pulsed laser deposition. J. Alloys Compd..

[72] Pearton S.J., Yang J., Cary P.H., Ren F., Kim J., Tadjer M.J., Mastro M.A. (2018). A review of Ga_2_O_3_ materials, processing, and devices. Appl. Phys. Rev..

[73] Chen Z., Wang X., Zhang F., Noda S., Saito K., Tanaka T., Nishio M., Guo Q. (2016). Temperature dependence of luminescence spectra in europium doped Ga_2_O_3_ film. J. Lumin..

[74] Wang Q., Chen J., Huang P., Li M., Lu Y., Homewood K.P., Chang G., Chen H., He Y. (2019). Influence of growth temperature on the characteristics of β-Ga_2_O_3_ epitaxial films and related solar-blind photodetectors. Appl. Surf. Sci..

[75] Yadav M.K., Mondal A., Das S., Sharma S., Bag A. (2019). Impact of annealing temperature on band-alignment of PLD grown Ga_2_O_3_/Si (100) heterointerface. J. Alloys Compd..

[76] Yu J., Nie Z., Dong L., Yuan L., Li D., Huang Y., Zhang L., Zhang Y., Jia R. (2019). Influence of annealing temperature on structure and photoelectrical performance of β-Ga_2_O_3_/4H-SiC heterojunction photodetectors. J. Alloys Compd..

[77] Puurunen R.L. (2014). A Short History of Atomic Layer Deposition: Tuomo Suntola’s Atomic Layer Epitaxy. Chem. Vap. Depos..

[78] Siah S.C., Brandt R.E., Lim K., Schelhas L.T., Jaramillo R., Heinemann M.D., Chua D., Wright J., Perkins J.D., Segre C.U. (2015). Dopant activation in Sn-doped Ga_2_O_3_ investigated by X-ray absorption spectroscopy. Appl. Phys. Lett..

[79] Choi D.-W., Chung K.-B., Park J.-S. (2013). Low temperature Ga_2_O_3_ atomic layer deposition using gallium tri-isopropoxide and water. Thin Solid Films.

[80] Mizutani F., Higashi S., Inoue M., Nabatame T. (2020). Atomic layer deposition of high purity Ga_2_O_3_ films using liquid pentamethylcyclopentadienyl gallium and combinations of H_2_O and O_2_ plasma. J. Vac. Sci. Technol. A.

[81] Ilhom S., Mohammad A., Shukla D., Grasso J., Willis B.G., Okyay A.K., Biyikli N. (2021). Low-Temperature As-Grown Crystalline β-Ga_2_O_3_ Films via Plasma-Enhanced Atomic Layer Deposition. ACS Appl. Mater. Interfaces.

[82] Jiao Y., Jiang Q., Meng J., Zhao J., Yin Z., Gao H., Zhang J., Deng J., Zhang X. (2021). Growth and characteristics of β-Ga_2_O_3_ thin films on sapphire (0001) by low pressure chemical vapour deposition. Vacuum.

[83] Tao J., Lu H.-L., Gu Y., Ma H.-P., Li X., Chen J.-X., Liu W.-J., Zhang H., Feng J.-J. (2019). Investigation of growth characteristics, compositions, and properties of atomic layer deposited amorphous Zn-doped Ga_2_O_3_ films. Appl. Surf. Sci..

[84] Sasaki K., Higashiwaki M., Kuramata A., Masui T., Yamakoshi S. (2013). MBE grown Ga_2_O_3_ and its power device applications. J. Cryst. Growth.

[85] Mazzolini P., Falkenstein A., Wouters C., Schewski R., Markurt T., Galazka Z., Martin M., Albrecht M., Bierwagen O. (2020). Substrate-orientation dependence of β-Ga_2_O_3_ (100), (010), (001), and (2¯01) homoepitaxy by indium-mediated metal-exchange catalyzed molecular beam epitaxy (MEXCAT-MBE). APL Mater..

[86] Nepal N., Katzer D.S., Downey B.P., Wheeler V.D., Nyakiti L.O., Storm D.F., Hardy M.T., Freitas J.A., Jin E.N., Vaca D. (2020). Heteroepitaxial growth of β-Ga_2_O_3_ films on SiC via molecular beam epitaxy. J. Vac. Sci. Technol. A Vac. Surf. Films.

[87] Kamimura T., Nakata Y., Higashiwaki M. (2021). Effect of (AlGa)_2_O_3_ back barrier on device characteristics of β-Ga_2_O_3_ metal-oxide-semiconductor field-effect transistors with Si-implanted channel. Jpn. J. Appl. Phys..

[88] Mazzolini P., Vogt P., Schewski R., Wouters C., Albrecht M., Bierwagen O. (2019). Faceting and metal-exchange catalysis in (010) β-Ga_2_O_3_ thin films homoepitaxially grown by plasma-assisted molecular beam epitaxy. APL Mater..

[89] Ngo T.S., Le D.D., Lee J., Hong S.-K., Ha J.-S., Lee W.-S., Moon Y.-B. (2020). Investigation of defect structure in homoepitaxial (2¯01) β-Ga_2_O_3_ layers prepared by plasma-assisted molecular beam epitaxy. J. Alloys Compd..

[90] Ahmadi E., Koksaldi O.S., Kaun S.W., Oshima Y., Short D.B., Mishra U.K., Speck J.S. (2017). Ge doping of β-Ga_2_O_3_ films grown by plasma-assisted molecular beam epitaxy. Appl. Phys. Express.

[91] Cheng Y., Xu Y., Li Z., Zhang J., Chen D., Feng Q., Xu S., Zhou H., Zhang J., Hao Y. (2020). Heteroepitaxial growth of α-Ga_2_O_3_ thin films on a-, c- and r-plane sapphire substrates by low-cost mist-CVD method. J. Alloys Compd..

[92] Kaneko K., Fujita S., Hitora T. (2018). A power device material of corundum-structured α-Ga_2_O_3_fabricated by MIST EPITAXY^®®^ technique. Jpn. J. Appl. Phys..

[93] Ma T., Chen X., Ren F., Zhu S., Gu S., Zhang R., Zheng Y., Ye J. (2019). Heteroepitaxial growth of thick α-Ga_2_O_3_ film on sapphire (0001) by MIST-CVD technique. J. Semicond..

[94] Morimoto S., Nishinaka H., Yoshimoto M. (2019). Growth and characterization of F-doped α-Ga_2_O_3_ thin films with low electrical resistivity. Thin Solid Films.

[95] Isomura N., Nagaoka T., Watanabe Y., Kutsuki K., Nishinaka H., Yoshimoto M. (2020). Determination of Zn-containing sites in β-Ga_2_O_3_ film grown through mist chemical vapor deposition via X-ray absorption spectroscopy. Jpn. J. Appl. Phys..

[96] Nishinaka H., Nagaoka T., Kajita Y., Yoshimoto M. (2021). Rapid homoepitaxial growth of (010) β-Ga_2_O_3_ thin films via mist chemical vapor deposition. Mater. Sci. Semicond. Process..

[97] Xu Y., Cheng Y., Li Z., Chen D., Xu S., Feng Q., Zhu W., Zhang Y., Zhang J., Zhang C. (2021). Ultrahigh-Performance Solar-Blind Photodetectors Based on High Quality Heteroepitaxial Single Crystalline β-Ga_2_O_3_ Film Grown by Vacuumfree, Low-Cost Mist Chemical Vapor Deposition. Adv. Mater. Technol..

[98] Lee S.-D., Kaneko K., Fujita S. (2016). Homoepitaxial growth of beta gallium oxide films by mist chemical vapor deposition. Jpn. J. Appl. Phys..

[99] Li Z., Jiao T., Yu J., Hu D., Lv Y., Li W., Dong X., Zhang B., Zhang Y., Feng Z. (2020). Single crystalline β-Ga_2_O_3_ homoepitaxial films grown by MOCVD. Vacuum.

[100] Feng Z., Bhuiyan A.F.M.A.U., Karim R., Zhao H. (2019). MOCVD homoepitaxy of Si-doped (010) β-Ga_2_O_3_ thin films with superior transport properties. Appl. Phys. Lett..

[101] Zhang Y., Alema F., Mauze A., Koksaldi O.S., Miller R., Osinsky A., Speck J.S. (2019). MOCVD grown epitaxial β-Ga_2_O_3_ thin film with an electron mobility of 176 cm2/V s at room temperature. APL Mater..

[102] Alema F., Hertog B., Osinsky A., Mukhopadhyay P., Toporkov M., Schoenfeld W.V. (2017). Fast growth rate of epitaxial β–Ga_2_O_3_ by close coupled showerhead MOCVD. J. Cryst. Growth.

[103] Li Z., Jiao T., Hu D., Lv Y., Li W., Dong X., Zhang Y., Feng Z., Zhang B. (2019). Study on β-Ga_2_O_3_ Films Grown with Various VI/III Ratios by MOCVD. Coatings.

[104] Tadjer M.J., Alema F., Osinsky A., Mastro M.A., Nepal N., Woodward J.M., Myers-Ward R.L., Glaser E.R., Freitas J.A., Jacobs A.G. (2020). Characterization of β-Ga_2_O_3_ homoepitaxial films and MOSFETs grown by MOCVD at high growth rates. J. Phys. D Appl. Phys..

[105] Alema F., Zhang Y., Osinsky A., Valente N., Mauze A., Itoh T., Speck J.S. (2019). Low temperature electron mobility exceeding 104 cm2/V s in MOCVD grown β-Ga_2_O_3_. APL Mater..

[106] Chikoidze E., Sartel C., Madaci I., Mohamed H., Vilar C., Ballesteros B., Belarre F., del Corro E., Castro P.V., Sauthier G. (2020). p-Type Ultrawide-Band-Gap Spinel ZnGa2O4: New Perspectives for Energy Electronics. Cryst. Growth Des..

[107] Chikoidze E., Rogers D., Teherani F., Rubio C., Sauthier G., Von Bardeleben H., Tchelidze T., Ton-That C., Fellous A., Bove P. (2019). Puzzling robust 2D metallic conductivity in undoped β-Ga_2_O_3_ thin films. Mater. Today Phys..

[108] Perez-Tomas A., Lira-Cantu M., Catalan G. (2016). Above-Bandgap Photovoltages in Antiferroelectrics. Adv. Mater..

[109] Pérez-Tomás A. (2019). Functional Oxides for Photoneuromorphic Engineering: Toward a Solar Brain. Adv. Mater. Interfaces.

[110] Chikoidze E., Von Bardeleben H.J., Akaiwa K., Shigematsu E., Kaneko K., Fujita S., Dumont Y. (2016). Electrical, optical, and magnetic properties of Sn doped α-Ga_2_O_3_ thin films. J. Appl. Phys..

[111] Guo D., Guo Q., Chen Z., Wu Z., Li P., Tang W. (2019). Review of Ga_2_O_3_-based optoelectronic devices. Mater. Today Phys..

[112] Goto K., Konishi K., Murakami H., Kumagai Y., Monemar B., Higashiwaki M., Kuramata A., Yamakoshi S. (2018). Halide vapor phase epitaxy of Si doped β-Ga_2_O_3_ and its electrical properties. Thin Solid Films.

[113] Yan X., Esqueda I.S., Ma J., Tice J., Wang H. (2018). High breakdown electric field in β-Ga_2_O_3_/graphene vertical barristor hetero-structure. Appl. Phys. Lett..

[114] Chikoidze E., Fellous A., Perez-Tomas A., Sauthier G., Tchelidze T., Ton-That C., Huynh T.T., Phillips M., Russell S., Jennings M. (2017). P-type β-gallium oxide: A new perspective for power and optoelectronic devices. Mater. Today Phys..

[115] Chikoidze E., Sartel C., Mohamed H., Madaci I., Tchelidze T., Modreanu M., Vales-Castro P., Rubio C., Arnold C., Sallet V. (2019). Enhancing the intrinsic p-type conductivity of the ultra-wide bandgap Ga_2_O_3_ semiconductor. J. Mater. Chem. C.

[116] Kyrtsos A., Matsubara M., Bellotti E. (2018). On the feasibility of p-type Ga_2_O_3_. Appl. Phys. Lett..

[117] Varley J.B., Janotti A., Franchini C., Van de Walle C. (2012). Role of self-trapping in luminescence andp-type conductivity of wide-band-gap oxides. Phys. Rev. B.

[118] Lyons J.L. (2018). A survey of acceptor dopants forβ-Ga_2_O_3_. Semicond. Sci. Technol..

[119] Sun D., Gao Y., Xue J., Zhao J. (2019). Defect stability and electronic structure of doped β-Ga_2_O_3_: A comprehensive ab initio study. J. Alloys Compd..

[120] Goyal A., Zakutayev A., Stevanović V., Lany S. (2021). Computational Fermi level engineering and doping-type conversion of Mg:Ga_2_O_3_ via three-step synthesis process. J. Appl. Phys..

[121] Sabino F.P., Cai X., Wei S.-H., Janotti A. (2019). Bismuth-Doped Ga_2_O_3_ as Candidate for p-Type Transparent Conducting Material. arXiv.

[122] Li L., Liao F., Hu X. (2020). The possibility of N–P codoping to realize P type β-Ga_2_O_3_. Superlattices Microstruct..

[123] Ma J., Lin J., Liu J., Li F., Liu Y., Yang G. (2020). Achieving high conductivity p-type Ga_2_O_3_ through Al-N and In-N co-doping. Chem. Phys. Lett..

[124] Zhang L., Yan J., Zhang Y., Li T., Ding X. (2012). A comparison of electronic structure and optical properties between N-doped β-Ga_2_O_3_ and N–Zn co-doped β-Ga_2_O_3_. Phys. B Condens. Matter.

[125] Qian Y., Guo D., Chu X., Shi H., Zhu W., Wang K., Huang X., Wang H., Wang S., Li P. (2017). Mg-doped p-type β-Ga_2_O_3_ thin film for solar-blind ultraviolet photodetector. Mater. Lett..

[126] Yue W., Yan J., Wu J., Zhang L. (2012). Structural and optical properties of Zn-doped β-Ga_2_O_3_films. J. Semicond..

[127] Alema F., Hertog B., Ledyaev O., Volovik D., Thoma G., Miller R., Osinsky A., Mukhopadhyay P., Bakhshi S., Ali H. (2017). Solar blind photodetector based on epitaxial zinc doped Ga2 O3 thin film. Phys. Status Solidi.

[128] Su Y., Guo D., Ye J., Zhao H., Wang Z., Wang S., Li P., Tang W. (2019). Deep Level Acceptors of Zn-Mg Divalent Ions Dopants in b-Ga_2_O_3_ for the Difficulty to p-Type Conductivity. J. Alloys Compd..

[129] Feng Q., Liu J., Yang Y., Pan D., Xing Y., Shi X., Xia X., Liang H. (2016). Catalytic growth and characterization of single crystalline Zn doped p-type β-Ga_2_O_3_ nanowires. J. Alloys Compd..

[130] Islam M., Liedke M.O., Winarski D., Butterling M., Wagner A., Hosemann P., Wang Y., Uberuaga B., Selim F.A. (2020). Chemical manipulation of hydrogen induced high p-type and n-type conductivity in Ga_2_O_3_. Sci. Rep..

[131] Wu Z., Jiang Z., Ma C., Ruan W., Chen Y., Zhang H., Zhang G., Fang Z., Kang J., Zhang T.-Y. (2021). Energy-driven multi-step structural phase transition mechanism to achieve high-quality p-type nitrogen-doped β-Ga_2_O_3_ films. Mater. Today Phys..

[132] Yao Y., Davis R.F., Porter L.M. (2017). Investigation of different metals as ohmic contacts to β-Ga_2_O_3_: Comparison and analysis of electrical behavior, morphology, and other physical properties. J. Electron. Mater..

[133] Ji M., Taylor N.R., Kravchenko I., Joshi P., Aytug T., Cao L.R., Paranthaman M.P. (2020). Demonstration of Large-Size Vertical Ga_2_O_3_ Schottky Barrier Diodes. IEEE Trans. Power Electron..

[134] Yao Y., Gangireddy R., Kim J., Das K.K., Davis R.F., Porter L.M. (2017). Electrical behavior of β-Ga_2_O_3_ Schottky diodes with different Schottky metals. J. Vac. Sci. Technol. B Nanotechnol. Microelectron. Mater. Process. Meas. Phenom..

[135] Lu X., Zhou L., Chen L., Ouyang X., Tang H., Liu B., Xu J. (2019). X-ray Detection Performance of Vertical Schottky Photodiodes Based on a Bulk β-Ga_2_O_3_ Substrate Grown by an EFG Method. ECS J. Solid State Sci. Technol..

[136] Higashiwaki M., Konishi K., Sasaki K., Goto K., Nomura K., Thieu Q.T., Togashi R., Murakami H., Kumagai Y., Monemar B. (2016). Temperature-dependent capacitance–voltage and current–voltage characteristics of Pt/Ga_2_O_3_ (001) Schottky barrier diodes fabricated on n^–^–Ga_2_O_3_ drift layers grown by halide vapor phase epitaxy. Appl. Phys. Lett..

[137] Harada T., Ito S., Tsukazaki A. (2019). Electric Dipole Effect in PdCoO_2_/β-Ga_2_O_3_ Schottky Diodes for High-Temperature Operation. Sci. Adv..

[138] Pérez-Tomás A., Mingorance A., Tanenbaum D., Lira-Cantú M., Lira-Cantu M. (2018). Chapter 8—Metal Oxides in Photovoltaics: All-Oxide, Ferroic, and Perovskite Solar Cells. The Future of Semiconductor Oxides in Next-Generation Solar Cells.

[139] Carey IV P.H., Yang J., Ren R., Sharma R., Law M., Pearton S.J. (2019). Comparison of Dual-Stack Dielectric Field Plates on β-Ga_2_O_3_ Schottky Rectifiers. ECS J. Solid State Sci. Technol..

[140] Chen Y.-T., Yang J., Ren F., Chang C.-W., Lin J., Pearton S.J., Tadjer M.J., Kuramata A., Liao Y.-T. (2019). Implementation of a 900 V Switching Circuit for High Breakdown Voltage β-Ga_2_O_3_ Schottky Diodes. ECS J. Solid State Sci. Technol..

[141] Hu Z., Zhou H., Feng Q., Zhang J., Zhang C., Dang K., Cai Y., Feng Z., Gao Y., Kang X. (2018). Field-Plated Lateral β-Ga_2_O_3_ Schottky Barrier Diode with High Reverse Blocking Voltage of More Than 3 kV and High DC Power Figure-of-Merit of 500 MW/cm^2^. IEEE Electron Device Lett..

[142] Hu Z., Zhou H., Dang K., Cai Y., Feng Z., Gao Y., Feng Q., Zhang J., Hao Y. (2018). Lateral β -Ga_2_O_3_ Schottky Barrier Diode on Sapphire Substrate With Reverse Blocking Voltage of 1.7 kV. IEEE J. Electron Devices Soc..

[143] Oh S., Yang G., Kim J. (2016). Electrical Characteristics of Vertical Ni/β-Ga_2_O_3_ Schottky Barrier Diodes at High Temperatures. ECS J. Solid State Sci. Technol..

[144] Müller S., Thyen L., Splith D., Reinhardt A., Wenckstern H.V., Grundmann M. (2019). High-Quality Schottky Barrier Diodes on β-Gallium Oxide Thin Films on Glass Substrate. ECS J. Solid State Sci. Technol.

[145] Tadjer M.J., Wheeler V.D., Shahin D.I., Eddy C.R., Kub F.J. (2017). Thermionic Emission Analysis of TiN and Pt Schottky Contacts to β-Ga_2_O_3_. ECS J. Solid State Sci. Technol..

[146] Du L., Xin Q., Xu M., Liu Y., Mu W., Yan S., Wang X., Xin G., Jia Z., Tao X.-T. (2019). High-Performance Ga_2_O_3_ Diode Based on Tin Oxide Schottky Contact. IEEE Electron Device Lett..

[147] Hu Z., Li J., Zhao C., Feng Z., Tian X., Zhang Y., Zhang Y., Ning J., Zhou H., Zhang C. (2020). Design and Fabrication of Vertical Metal/TiO2/β-Ga_2_O_3_ Dielectric Heterojunction Diode With Reverse Blocking Voltage of 1010 V. IEEE Trans. Electron Devices.

[148] Fontserè A., Pérez-Tomás A., Banu V., Godignon P., Millán J., de Vleeschouwer H., Parsey J.M., Moens P. (2012). A HfO2 based 800V/300 °C Au-Free AlGaN/GaN-on-Si HEMT Technology. Proceedings of the 2012 24th International Symposium on Power Semiconductor Devices and ICs.

[149] Zhou H., Yan Q.L., Zhang J.C., Lv Y.J., Liu Z.H., Zhang Y.N., Dang K., Dong P.F., Feng Z.Q., Feng Q. (2019). High-Performance Vertical β-Ga_2_O_3_ Schottky Barrier Diode With Implanted Edge Termination. IEEE Electron Device Lett..

[150] Lin C.-H., Yuda Y., Wong M.H., Sato M., Takekawa N., Konishi K., Watahiki T., Yamamuka M., Murakami H., Kumagai Y. (2019). Vertical Ga_2_O_3_ Schottky Barrier Diodes With Guard Ring Formed by Nitrogen-Ion Implantation. IEEE Electron Device Lett..

[151] Wang Y.G., Lv Y.J., Long S.B., Zhou X.Y., Song X.B., Liang S.L., Han T.T., Tan X., Feng Z.H., Cai S.J. (2020). High-Voltage (201) β-Ga_2_O_3_ Vertical Schottky Barrier Diode With Thermally-Oxidized Termination. IEEE Electron Device Lett..

[152] Allen N., Xiao M., Yan X., Sasaki K., Tadjer M.J., Ma J., Zhang R., Wang H., Zhang Y. (2019). Vertical Ga_2_O_3_ Schottky Barrier Diodes With Small-Angle Beveled Field Plates: A Baliga’s Figure-of-Merit of 0.6 GW/cm^2^. IEEE Electron Device Lett..

[153] Li W.S., Nomoto K., Hu Z.Y., Jena D., Xing H.L.G. (2020). Field-Plated Ga_2_O_3_ Trench Schottky Barrier Diodes With a BV2/R_on,sp_ of up to 0.95 GW/cm^2^. IEEE Electron Device Lett..

[154] Saitoh Y., Sumiyoshi K., Okada M., Horii T., Miyazaki T., Shiomi H., Ueno M., Katayama K., Kiyama M., Nakamura T. (2010). Extremely Low On-Resistance and High Breakdown Voltage Observed in Vertical GaN Schottky Barrier Diodes with High-Mobility Drift Layers on Low-Dislocation-Density GaN Substrates. Appl. Phys. Express.

[155] Xiao M., Ma Y., Cheng K., Liu K., Xie A., Beam E., Cao Y., Zhang Y. (2020). 3.3 kV Multi-Channel AlGaN/GaN Schottky Barrier Diodes With P-GaN Termination. IEEE Electron Device Lett..

[156] Kizilyalli I.C., Edwards A.P., Aktas O., Prunty T., Bour D. (2014). Vertical Power p-n Diodes Based on Bulk GaN. IEEE Trans. Electron Devices.

[157] Watahiki T., Yuda Y., Furukawa A., Yamamuka M., Takiguchi Y., Miyajima S. (2017). Heterojunction p-Cu2O/n-Ga_2_O_3_ diode with high breakdown voltage. Appl. Phys. Lett..

[158] Lu X., Zhou X., Jiang H., Ng K.W., Chen Z., Pei Y., Lau K.M., Wang G. (2020). 1-kV Sputtered p-NiO/n-Ga_2_O_3_ Heterojunction Diodes with an Ultra-Low Leakage Current Below 1uA/cm2. IEEE Electron Device Lett..

[159] Gong H.H., Chen X.H., Xu Y., Ren F.-F., Gu S.L., Ye J.D. (2020). A 1.86-kV Double-Layered NiO/β-Ga_2_O_3_ Vertical p–n Heterojunction Diode. Appl. Phys. Lett..

[160] Pérez-Tomás A., Chikoidze E., Dumont Y., Jennings M.R., Russell S.O., Vales-Castro P., Catalan G., Lira-Cantú M., Ton–That C., Teherani F.H. (2019). Giant Bulk Photovoltaic Effect in Solar Cell Architectures with Ultra-Wide Bandgap Ga_2_O_3_ Transparent Conducting Electrodes. Mater. Today Energy.

[161] Russell S.A.O., Jennings M.R., Dai T.X., Li F., Hamilton D.P., Fisher C.A., Sharma Y.K., Mawby P.A., Pérez-Tomás A. (2017). Functional Oxide as an Extreme High-k Dielectric towards 4H-SiC MOSFET Incorporation. Mater. Sci. Forum.

[162] Xia Z., Chandrasekar H., Moore W., Wang C., Lee A.J., McGlone J., Kalarickal N.K., Arehart A., Ringel S., Yang F. (2019). Metal/BaTiO_3_/β-Ga_2_O_3_ dielectric heterojunction diode with 5.7 MV/cm breakdown field. Appl. Phys. Lett..

[163] Razzak T., Chandrasekar H., Hussain K., Lee C.H., Mamun A., Xue H., Xia Z., Sohel S.H., Rahman M.W., Bajaj S. (2020). BaTiO_3_/Al0.58Ga0.42N lateral heterojunction diodes with breakdown field exceeding 8 MV/cm. Appl. Phys. Lett..

[164] Kalarickal N.K., Feng Z., Bhuiyan A., Xia Z., McGlone J.F., Moore W., Arehart A.R., Ringel S.A., Zhao H., Rajan S. (2020). Electrostatic engineering using extreme permittivity materials for ultra-wide band gap semiconductor transistors. arXiv.

[165] Roy S., Bhattacharyya A., Krishnamoorthy S. (2020). Analytical Modeling and Design of Gallium Oxide Schottky Barrier Diodes Beyond Unipolar Figure of Merit Using High-k Dielectric Superjunction Structures. arXiv.

[166] Pérez-Tomás A., Fontserè A., Jennings M.R., Gammon P.M. (2013). Modeling the Effect of Thin Gate Insulators (SiO_2_, SiN, Al_2_O_3_ and HfO2) on AlGaN/GaN HEMT Forward Characteristics Grown on Si, Sapphire and SiC. Mater. Sci. Semicond. Processing.

[167] Sasaki K., Higashiwaki M., Kuramata A., Masui T., Yamakoshi S. (2013). Si-Ion Implantation Doping in β-Ga_2_O_3_and Its Application to Fabrication of Low-Resistance Ohmic Contacts. Appl. Phys. Express.

[168] Zhou H., Si M., Alghamdi S., Qiu G., Yang L., Ye P.D. (2017). High performance depletion/enhancement-mode β-Ga_2_O_3_ on insulator (GOOI) field-effect transistors with record drain currents of 600/450 mA/mm. IEEE Electron Device Lett..

[169] Carey P.H., Yang J., Ren F., Hays D.C., Pearton S.J., Jang S., Kuramata A., Kravchenko I.I. (2017). Ohmic contacts on n-type β-Ga_2_O_3_ using AZO/Ti/Au. AIP Adv..

[170] Fontserè A., Pérez-Tomás A., Placidi M., Fernández-Martínez P., Baron N., Chenot S., Cordier Y., Moreno J., Gammon P., Jennings M. (2011). Temperature dependence of Al/Ti-based Ohmic contact to GaN devices: HEMT and MOSFET. Microelectron. Eng..

[171] Fontserè A., Pérez-Tomás A., Placidi M., Llobet J., Baron N., Chenot S., Cordier Y., Moreno J.C., Gammon P.M., Jennings M.R. (2011). Micro and Nano Analysis of 0.2 Ω mm Ti/Al/Ni/Au Ohmic Contact to AlGaN/GaN. Appl. Phys. Lett..

[172] Li Z., Liu Y., Zhang A., Liu Q., Shen C., Wu F., Xu C., Chen M., Fu H., Zhou C. (2018). Quasi-two-dimensional β-Ga_2_O_3_ field effect transistors with large drain current density and low contact resistance via controlled formation of interfacial oxygen vacancies. Nano Res..

[173] Chabak K.D., McCandless J.P., Moser N.A., Green A.J., Mahalingam K., Crespo A., Hendricks N., Howe B.M., Tetlak S.E., Leedy K. (2018). Recessed-gate enhancement-mode-Ga_2_O_3_ MOSFETs. IEEE Electron Device Lett..

[174] Hu Z., Nomoto K., Li W., Tanen N., Sasaki K., Kuramata A., Nakamura T., Jena D., Xing H.G. (2018). Enhancement-Mode Ga_2_O_3_ Vertical Transistors With Breakdown Voltage >1 kV. IEEE Electron Device Lett..

[175] Hu Z., Nomoto K., Li W., Jinno R., Nakamura T., Jena D., Xing H. (2019). 1.6 kV Vertical Ga_2_O_3_ FinFETs With Source-Connected Field Plates and Normally-off Operation. Proceedings of the 31st International Symposium on Power Semiconductor Devices and ICs (ISPSD).

[176] Li W., Nomoto K., Hu Z., Nakamura T., Jena D., Xing H.G. Single and multi-fin normally-off Ga_2_O_3_ vertical transistors with a breakdown voltage over 2.6 kV. Proceedings of IEDM Technical Digest.

[177] Lv Y., Zhou X., Long S., Song X., Wang Y., Liang S., He Z., Han T., Tan X., Feng Z. (2019). Source-Field-Plated β-Ga_2_O_3_ MOSFET with Record Power Figure of Merit of 50.4 MW/cm^2^. IEEE Electron Device Lett..

[178] Lv Y., Liu H., Zhou X., Wang Y., Song X., Cai Y., Yan Q., Wang C., Liang S., Zhang J. (2020). Lateral β-Ga_2_O_3_ MOSFETs With High Power Figure of Merit of 277 MW/cm^2^. IEEE Electron Device Lett..

[179] Tetzner K., Treidel E.B., Hilt O., Popp A., Anooz S.B., Wagner G., Thies A., Ickert K., Gargouri H., Würfl J. (2019). Lateral 1.8 kV β-Ga_2_O_3_ MOSFET with 155 MW/cm^2^ Power Figure of Merit. IEEE Electron Device Lett..

[180] Sharma S., Zeng K., Saha S., Singisetti U. (2020). Field-plated lateral Ga_2_O_3_ MOSFETs with polymer passivation and 8.03 kV breakdown voltage. IEEE Electron Device Lett..

[181] Shibata D., Kajitani R., Ogawa M., Tanaka K., Tamura S., Hatsuda T. 1.7 kV/1.0 mΩcm^2^ normally-off vertical GaN transistor on GaN substrate with regrown p-GaN/AlGaN/GaN semipolar gate structure. Proceedings of IEDM Technical Digest.

[182] Uemoto Y., Shibata D., Yanagihara M., Ishida H., Matsuo H., Nagai S. 8300 V blocking voltage AlGaN/GaN power HFET with thick poly-AlN passivation. Proceedings of IEEE International Electron Devices Meeting.

[183] Zhang Y., Neal A., Xia Z., Joishi C., Johnson J.M., Zheng Y., Bajaj S., Brenner M., Dorsey D., Chabak K. (2018). Demonstration of high mobility and quantum transport in modulation-doped β-(AlxGa1-x)_2_O_3_/Ga_2_O_3_ heterostructures. Appl. Phys. Lett..

[184] Song K., Zhang H., Fu H., Yang C., Singh R., Zhao Y., Sun H., Long S. (2020). Normally-off AlN/β-Ga_2_O_3_ field-effect transistors using polarization-induced doping. J. Phys. D Appl. Phys..

[185] Lyons J.L. (2019). Electronic Properties of Ga_2_O_3_ Polymorphs. ECS J. Solid State Sci. Technol..

[186] Roy R., Hill V.G., Osborn E.F. (1952). Polymorphism of Ga_2_O_3_ and the System Ga_2_O_3_—H_2_O. J. Am. Chem. Soc..

[187] Oshima T., Okuno T., Arai N., Kobayashi Y., Fujita S. (2009). β-Al_2x_Ga_2-2x_O_3_ Thin Film Growth by Molecular Beam Epitaxy. Jpn. J. Appl. Phys..

[188] Jinno R., Chang C.S., Onuma T., Cho Y., Ho S.-T., Rowe D., Cao M.C., Lee K., Protasenko V., Schlom D.G. (2021). Crystal orientation dictated epitaxy of ultrawide-bandgap 5.4- to 8.6-eV α-(AlGa)_2_O_3_ on m-plane sapphire. Sci. Adv..

[189] Jinno R., Kaneko K., Fujita S. (2021). Thermal stability of α-(Al_x_Ga_1–x_)_2_O_3_ films grown on c-plane sapphire substrates with an Al composition up to 90%. Jpn. J. Appl. Phys..

[190] Chi Z., Tarntair F.-G., Frégnaux M., Wu W.-Y., Sartel C., Madaci I., Chapon P., Sallet V., Dumont Y., Pérez-Tomás A. (2021). Bipolar Self-doping in Ultra-wide Bandgap Spinel ZnGa_2_O_4_. Mater. Today Phys..

[191] Chen M.-I., Singh A., Chiang J.-L., Horng R.-H., Wuu D.-S. (2020). Zinc Gallium Oxide—A Review from Synthesis to Applications. Nanomaterials.

[192] Galazka Z., Irmscher K., Pietsch M., Ganschow S., Schulz D., Klimm D., Hanke I.M., Schroeder T., Bickermann M. (2021). Experimental Hall electron mobility of bulk single crystals of transparent semiconducting oxides. J. Mater. Res..

[193] Galazka Z., Klimm D., Irmscher K., Uecker R., Pietsch M., Bertram R., Naumann M., Albrecht M., Kwasniewski A., Schewski R. (2015). MgGa_2_O_4_ as a new wide bandgap transparent semiconducting oxide: Growth and properties of bulk single crystals. Phys. Status Solidi.

